# Identification of the Prognostic Signatures of Glioma With Different *PTEN* Status

**DOI:** 10.3389/fonc.2021.633357

**Published:** 2021-07-14

**Authors:** Pei Zhang, Xinyi Meng, Liqun Liu, Shengzhen Li, Yang Li, Sakhawat Ali, Shanhu Li, Jichuan Xiong, Xuefeng Liu, Shouwei Li, Qin Xia, Lei Dong

**Affiliations:** ^1^ School of Life Science, Beijing Institute of Technology, Beijing, China; ^2^ Department of Cell Engineering, Beijing Institute of Biotechnology, Beijing, China; ^3^ School of Electronic and Optical Engineering, Nanjing University of Science and Technology, Nanjing, China; ^4^ Beijing Sanbo Brain Hospital, Capital Medical University, Beijing, China

**Keywords:** glioma, mutant PTEN, prognostic risk model, risk score, prognostic signature

## Abstract

The high-grade glioma is characterized by cell heterogeneity, gene mutations, and poor prognosis. The deletions and mutations of the tumor suppressor gene *PTEN* (5%-40%) in glioma patients are associated with worse survival and therapeutic resistance. Characterization of unique prognosis molecular signatures by *PTEN* status in glioma is still unclear. This study established a novel risk model, screened optimal prognostic signatures, and calculated the risk score for the individual glioma patients with different *PTEN* status. Screening results revealed fourteen independent prognostic gene signatures in PTEN-wt and three in the -50PTEN-mut subgroup. Moreover, we verified risk score as an independent prognostic factor significantly correlated with tumor malignancy. Due to the higher malignancy of the PTEN-mut gliomas, we explored the independent prognostic signatures (*CLCF1*, *AEBP1*, and *OS9*) for a potential therapeutic target in PTEN-mut glioma. We further separated IDH wild-type glioma patients into GBM and LGG to verify the therapeutic target along with PTEN status, notably, the above screened therapeutic targets are also significant prognostic genes in both IDH-wt/PTEN-mut GBM and LGG patients. We further identified the small molecule compound (+)-JQ1 binds to all three targets, indicating a potential therapy for PTEN-mut glioma. In sum, gene signatures and risk scores in the novel risk model facilitate glioma diagnosis, prognosis prediction, and treatment.

## Introduction

The most common primary brain tumor, glioma, starts with glial support cells around nerve cells (1, 2). Clinically, glioma is associated with high mortality and recurrence rates and poor prognosis (3, 4). Although surgery, radiotherapy, and alkylated chemotherapies are used in treatment, patient survival rate did not significantly improve in the past decade (5). Genomics, transcriptomics, and epigenetic analyses gave rise to new concepts for the molecular classification and treatment of gliomas (6). Thus, the molecular signature is important for the tumor’s diagnosis, patient stratification, and personalized treatment. In 2016, the World Health Organization (WHO) incorporated morphology and genetic variations to update guidelines for classifying brain tumors, particularly gliomas (7). Many studies have shown that glioma patients with IDH (Isocitrate dehydrogenase) mutations have a better prognosis (8–14). Thus, the WHO included IDH mutation as an essential diagnostic and classification criterion for glioma (7). Additionally, MGMT (O6-methylguanine-DNA methyltransferase) promoter methylation is another example of a glioma signature. MGMT was initially identified as a prognostic and predictive signature for glioma diagnosis in patients treated with temozolomide (15). Therefore, signatures in glioma have been highly valued as prognostic, predictive, and clinical application targets.

WHO divided glioma into grades I, II, III, and IV. The higher the glioma grade, the worst the prognosis (16–19). Even though grade IV glioma (glioblastoma multiforme, GBM) is considered different from low-grade glioma (LGG) in general, many studies still combine LGG and GBM dataset to screen tumorigenesis marker or therapeutic target by considering GBM is the most malignant form of glioma (6, 20). Notably, 50% of GBM harbor somatic alterations in the phosphatidylinositol-3-kinase (PI3K) pathway (21, 22). The tumor suppressor PTEN is the most critical negative regulator of the PI3K pathway (23). PTEN is significantly altered in GBMs (30–40%), and mutational loss of PTEN function is an important malignancy event in glioma. The meta-analysis by Han et al. showed that *PTEN* mutations are strongly associated with shorter survival in glioma patients, suggesting that *PTEN* status strongly correlates with the prognosis of patients (24). Additionally, the loss of PTEN increases drug resistance. For example, (1) GBM patients with *PTEN* mutation have no significant response to anti-PD-1 (Programmed cell death 1) immunotherapy due to the mutation-induced changes in the immune microenvironment (25); (2) patients with PTEN-negative GBM have a shorter survival time after initiation of bevacizumab than those with PTEN-positive GBM (26); (3) loss of PTEN leads to clinical resistance to PI3Kα inhibitors (27); (4) fibroblast growth factor receptor 2-mediated phosphorylation of PTEN at tyrosine 240 contributes to the radioresistance of glioma (28).

Detecting the *PTEN* status of glioma patients and treating them separately according to their unique signatures may reduce drug resistance and improve the survival of patients. In this study, we divided glioma patients into PTEN-wt and PTEN-mut subgroups. We observed that survival of the PTEN-mut is significantly lower than the PTEN-wt subgroup in both TCGA and the CGGA glioma datasets, indicating the correlation between *PTEN* status and the prognosis of gliomas patients. We established prognostic risk models by fitting the L1-penalized (LASSO) Cox-PH regression model, obtained the optimal prognostic signatures, and calculated risk scores in these two glioma subgroups. Due to the higher malignancy of the PTEN-mut glioma, we look for potential therapies targeting the prognostic signatures (*CLCF1*, *AEBP1*, and *OS9*) in this subgroup. We then focused on IDH-wt/PTEN-mut GBM and LGG patients and found that the aforementioned screened genes also significantly prognostic in both subgroups. Finding tissue/cancer-specific miRNAs, TFs (transcription factors), and the small molecular compound is critical in the tumor treatment (29–33). Herein, we provide some miRNAs and TFs binding to the optimal prognostic genes, and small molecular compounds binding to the respective proteins in the PTEN-mut subgroup. Importantly, we found that (+)-JQ1 compound interacting with these three optimal prognostic genes as a potential drug.

## Materials and Methods

### Data Source

The Cancer Genome Atlas Program (TCGA) glioma project is the training dataset. The RNA-seq data was downloaded from https://tcga.xenahubs.net/download/TCGA.GBMLGG.sampleMap/HiSeqV2.gz, including 697 glioma samples and 5 Normal samples. This data, which combines TCGA brain lower grade glioma and glioblastoma multiforme datasets, shows the gene-level transcription estimates, as in log2 (x+1) transformed RSEM Normalized count. The clinical information of 1148 patients was downloaded from https://tcga.xenahubs.net/download/TCGA.GBMLGG.sampleMap/GBMLGG_clinicalMatrix. Finally, the mutation data was download from https://gdc.xenahubs.net/download/TCGA-LGG.varscan2_snv.tsv.gz and https://gdc.xenahubs.net/download/TCGA-GBM.varscan2_snv.tsv.gz, including 506 low-grade gliomas samples and 390 glioblastomas samples, respectively. Two types of somatic mutations in the dataset are single nucleotide polymorphisms (SNPs) and small insertion/deletions (INDELs). Each patient must have clinical, RNA-seq, and mutation information; thus, the 653 overlapping patients in three data is our training and research dataset (**Supplementary Table 1**).

The Chinese Glioma Genome Atlas (CGGA) glioma project is the validation dataset. The RNA-seq dataset of CGGA was download from http://cgga.org.cn/download?file=download/20200506/CGGA.mRNAseq_693.RSEM-genes.20200506.txt.zip&type=mRNAseq_693&time=20200506 (expression data from STAR+RSEM), and corresponding clinical information was downloaded from http://cgga.org.cn/download?file=download/20200506/CGGA.mRNAseq_693_clinical.20200506.txt.zip&type=mRNAseq_693_clinical&time=20200506, which contains 693 glioma samples. The mutation dataset was download from http://cgga.org.cn/download?file=download/20200506/CGGA.WEseq_286.20200506.txt.zip&type=WEseq_286&time=20200506, containing 286 glioma samples. A total of 144 overlapping patient samples from the three datasets were used as a validation dataset (**Supplementary Table 1**).

### Differentially Expressed Genes

**Figure 1:**
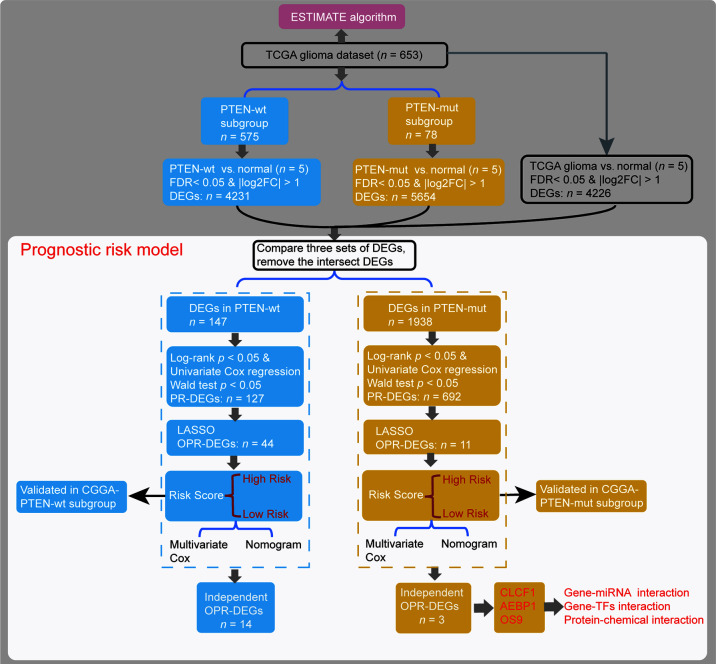
Data analysis flow *(Materials and Methods)*.

Patients were divided into two glioma subgroups according to the status of PTEN (**Figure 1**), including PTEN-wt and PTEN-mut patients, whose clinical features are respectively presented in **Table 1**. Differentially expressed genes (DEGs) between gliomas of our dataset and Normal samples (DEGs-all from TCGA glioma *vs.* Normal, 653 *vs.* 5; DEGs-wt from TCGA PTEN-wt *vs.* Normal, 575 *vs.* 5; DEGs-mut from TCGA PTEN-mut *vs.* Normal, 78 *vs.* 5) were screened (**Figure 1**) using the *limma* package (version 3.40.6) of R 3.6.1 (34). Fold discovery rate (FDR) < 0.05 and |log2FC (fold change) | > 1 was as the selection criteria. Based on the expression values of the DEGs, the clustering analysis was used by *heatmap* (version 1.0.12) (35).

**Table 1:** Clinical information of the PTEN-wt and PTEN-mut subgroups (*** significant difference).

Group	PTEN_wt	PTEN_mut	*p-value*
Patient(number)	575	78	
Age(median age)	44	59	<0.0001***
OS.time(median days)	576	461	<0.0001***
Male	57% (330)	59% (44)	
Female	43% (245)	44% (34)	
Chemotherapy	19% (108)	14% (4)	
Radiation therapy	9% (50)	3% (1)	
Astrocytoma	30% (171)	30% (24)	
Oligoastrocytoma	22% (124)	14% (11)	
Oligodendroglioma	31% (180)	8% (6)	
Glioblastoma	17% (100)	63% (49)	
IDH1_mutation	20% (116)	12% (9)	

### Prognostic Risk Models

We established prognostic risk models using DEGs in different *PTEN* statuses and machine learning. In brief, we obtained prognostic DEGs (PR-DEGs) that were significantly linked to survival time by calculating the log-rank test and univariate Cox regression (**Figure 1**). A gene with log-rank *p* < 0.05 and Wald test *p* < 0.05 was considered as a significant PR-DEG. These identified PR-DEGs were then used to fit L1-penalized (LASSO) Cox-PH regression model (35) for the selection of the optimal prognostic DEGs (OPR-DEGs) with *glmnet* package (version 3.0.2) of R (**Figure 1**). The optimal parameter of ‘lambda’ in the selection model was calculated *via* the cross-validation likelihood (CVL) method 1000 times.

A prognostic risk model is established from the DEGs’ expression levels and Cox regression coefficients (**Figure 1**). The risk score of every patient was established by the following formula (36):

Expression Risk Score=∑βRNAn×ExpRNAn

β_RNAn_ and Exp_RNAn_ represent the Cox-PH coefficient and the RNA expression level of ORP-DEGs.

Each subgroup set was classified into high-risk patients and low-risk patients by median risk score. After that, the Kaplan-Meier method was used to draw the survival curves between subgroups. Additionally, the accuracy for survival prediction using these models was evaluated by the area under the receiver operating characteristic curve (AUC). The CGGA dataset was used to validate the prognostic risk model (**Figure 1**).

### Multivariate Cox Regression Analysis and Nomogram Survival Rate Model

Risk scores and clinical features were used for Multivariate Cox regression analysis (*survival* package, 3.1-8 in R) and Nomogram survival rate model (*rms* package, 5.1-4 in R) (37), to construct a nomogram 1-year, 3-year, 5-year, 10-year, and 15-year survival rate model (**Figure 1**). Bootstrap resamples (1000) in the TCGA dataset were used for internal validation, and the CGGA dataset was used for external validation to evaluate the predictive effect. The value of the C-index indicates the accuracy of the predictive ability.

### Generation of ESTIMATEScore and Tumor Purity Score

The ESTIMATE algorithm loaded in the estimate package (1.0.13) in R is used to calculate the ratio of the immune/stromal component in the tumor microenvironment, exhibited by four kinds of scores: Immune Score, Stromal Score, ESTIMATEScore, and TumorPurityScore (**Figure 1**).

### Function Analysis, Protein-Protein Interaction, Gene-miRNA and TFs Interaction and Protein-Chemical Interaction

In function analysis using the *clusterProfiler* package (version 3.12.0) in R, we analyzed the Kyoto Encyclopedia of Genes and Genomes (KEGG) pathway enrichment analyses for identified DEGs (*p* < 0.05). The protein-protein interactions (PPIs) of OPR-DEGs were analyzed based on the STRING database (Version: 11.0) (38). The gene-miRNA, gene-TFs, and protein–chemical interactions were constructed by NetworkAnalyst (39) and visualized using Cytoscape (Version 3.6.0) (40).

### Dock Between Proteins With Small Molecular Compound

Autodock (Version 4.2) was used to dock proteins and small molecular compounds, and VDM (Version 1.9.3) was used to visualize docking sites.

### Cell Culture

Colony formation experiment was used to analyze the effect of (+)-JQ1 on GBM cell proliferation. Cells (1000/well) of U251 (GBM cell line, PTEN-deficiency) and U343 (GBM cell line, PTEN-wt) were seeded into 12-well plates. After overnight culture, fresh medium with different concentrations of (+)-JQ1 was replaced. After 2 weeks of treatment, the medium was pumped, the cells were flushed with PBS 3 times, 2ml 4% paraformaldehyde was added to fix the cells (10 minutes), and the cells were washed with PBS for 3 times. After dyeing with 0.5% crystal violet for 10 minutes, colony formed.

## Results

### The PTEN-mut With Worse Survival Than the PTEN-wt Subgroup

**Figure 2:**
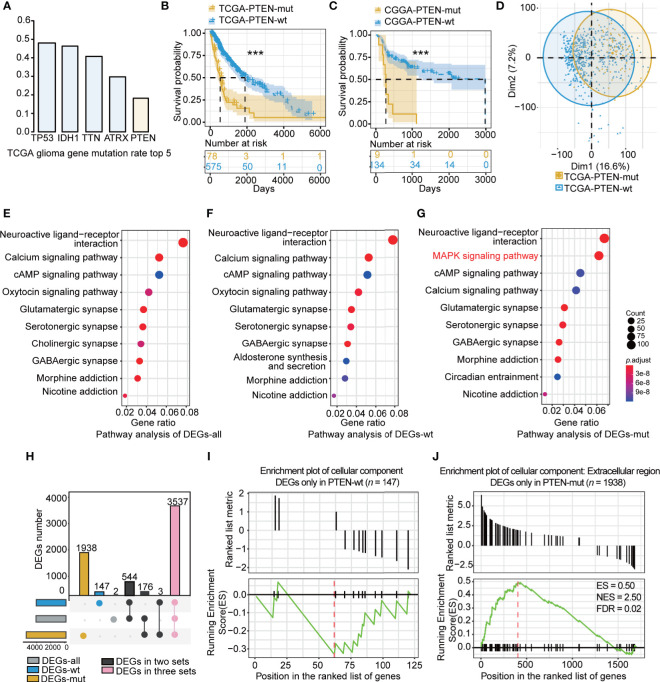
The characteristic of PTEN-wt and PTEN-mut subgroup: **(A)** The top 5 gene mutation rate in TCGA glioma; **(B)** Survival analysis (Kaplan Meier Curve) of PTEN-wt (blue, n = 575) and PTEN-wt subgroup (yellow, *n* = 78) in TCGA dataset; **(C)** Survival analysis (Kaplan Meier Curve) of PTEN-wt (blue, *n* = 134) and PTEN-wt subgroup (yellow, *n* = 9) in CGGA dataset; **(D)** The principal component analysis of TCGA PTEN-wt (blue, *n* = 575) and TCGA PTEN-mut subgroup (yellow, *n* = 78 ); **(E)** Pathway analysis of DEGs-all (DEGs from TCGA glioma vs. normal tissue, 653 vs 5); **(F)** Pathway analysis of DEGs-wt (DEGs from TCGA PTEN-wt glioma vs. normal tissue, 575 vs 5); **(G)** Pathway analysis of DEGs-mut (DEGs from TCGA PTEN-mut glioma vs. normal tissue, 78 vs 5); **(H)** The overlap of DEGs-all, DEGs-wt, and DEGs-mut; **(I)** The GESA enrichment plot of cellular component for DEGs only in PTEN-wt (*n* = 147); **(J)** The GESA enrichment plot of cellular component for DEGs only in PTEN-mut (*n* =1938). DEGs-all: DEGs from TCGA glioma vs. normal tissue ; DEGs-wt: DEGs from TCGA PTEN-wt glioma vs. normal tissue; DEGs-mut: DEGs from TCGA PTEN-mut glioma vs. normal tissue. ***p < 0.001.

The PTEN-mut group presented the worst survival. The TCGA dataset showed an 18% *PTEN* mutation rate in glioma (**Figure 2A**). Of the top five mutation genes in glioma, including *TP53*, *IDH1*, *TTN*, *ATRX*, and *PTEN*, the *PTEN* mutation is the most significant in reducing survival (**Supplementary Figures 1A–D** and **Figure 2B**). As expected, the CGGA dataset showed worse survival in PTEN-mut gliomas compared to the PTEN-wt subgroup (**Figure 2C**). To identify survival differences among mutated *PTEN* glioma patients, we extracted the PTEN mutation sites list (**Supplementary Table 1**) of TCGA glioma patients. The survival rates of the glioma patients with loss of function mutation (57%) and patients with non-reported mutation sites (43%) have no difference (**Supplementary Figure 1E**). Specifically, there was no significant difference (*p* = 0.78) in survival time between the truncated PTEN protein at the arginine 130 (R130, Complete loss of function; median survival time: 345; *n* = 4) and point mutation glutamine (R130Q, Partial loss of function; median survival time: 360; *n* = 3).

The clinical information of the PTEN-wt and the PTEN-mut subgroups was statistically analyzed (**Table 1**). The median age of the PTEN-wt subgroup was 44, and the PTEN-mut subgroup was 59, showing a significant statistical difference between the two subgroups (**Table 1**). In addition, PTEN-wt contains 17% GBM, while the poorer survival PTEN-mut subgroup includes 63% GBM, indicating that malignant GBM is more prone to *PTEN* mutation (**Table 1**). Thus, all data show that PTEN is a critical indicator of glioma prognosis.

Patients in different subgroups showed significant differences in gene expression as indicated by the principal component analysis (**Figure 2D**). To identify the subgroup-specific DEGs of the PTEN-wt and PTEN-mut subgroups, the DEGs-all, DEGs-wt, and DEGs-mut (see Materials and methods) were calculated (**Figure 1**). Notably, the pathway analysis of DEGs (**Figures 2E–G**) showed the enriched mitogen-activated protein kinase (MAPK) signaling pathway in the PTEN-mut subgroup (**Figure 2G**). MAPK signaling pathway is a set of the evolution of conservative serine/threonine-protein kinase, which controls many physiological activities, such as inflammation, apoptosis, canceration, invasion, and metastasis of tumor cells (41). The intersection of DEGs-all, DEGs-wt, and DEGs-mut in the distribution diagram showed that there were 3537 overlapping DEGs in both subgroups. However, 147 PTEN-wt group-specific DEGs and 1938 PTEN-mut subgroup-specific DEGs were also found (**Figure 2H**, **Supplementary Tables 3** and **4**), suggesting gliomas with *PTEN* mutation are non-homeostatic. Gene set enrichment analysis (GSEA) showed 147 DEGs were mainly down-regulated only in DEGs-wt (**Figure 2I**, **Supplementary Table 4**) without significant enrichment in the Cellular Component term. Notably, most of the 1938 DEGs were upregulated only in DEGs-mut (**Figure 2J**, **Supplementary Table 3**), having significant enrichment in the extracellular region of Cellular Component term, which is closely related to tumor progression (**Figure 2J**, FDR < 0.05). All of these results indicate that the PTEN-mut glioma presents a more malignant state.

### The Signature and Risk Score in the PTEN-wt Subgroup

**Figure 3:**
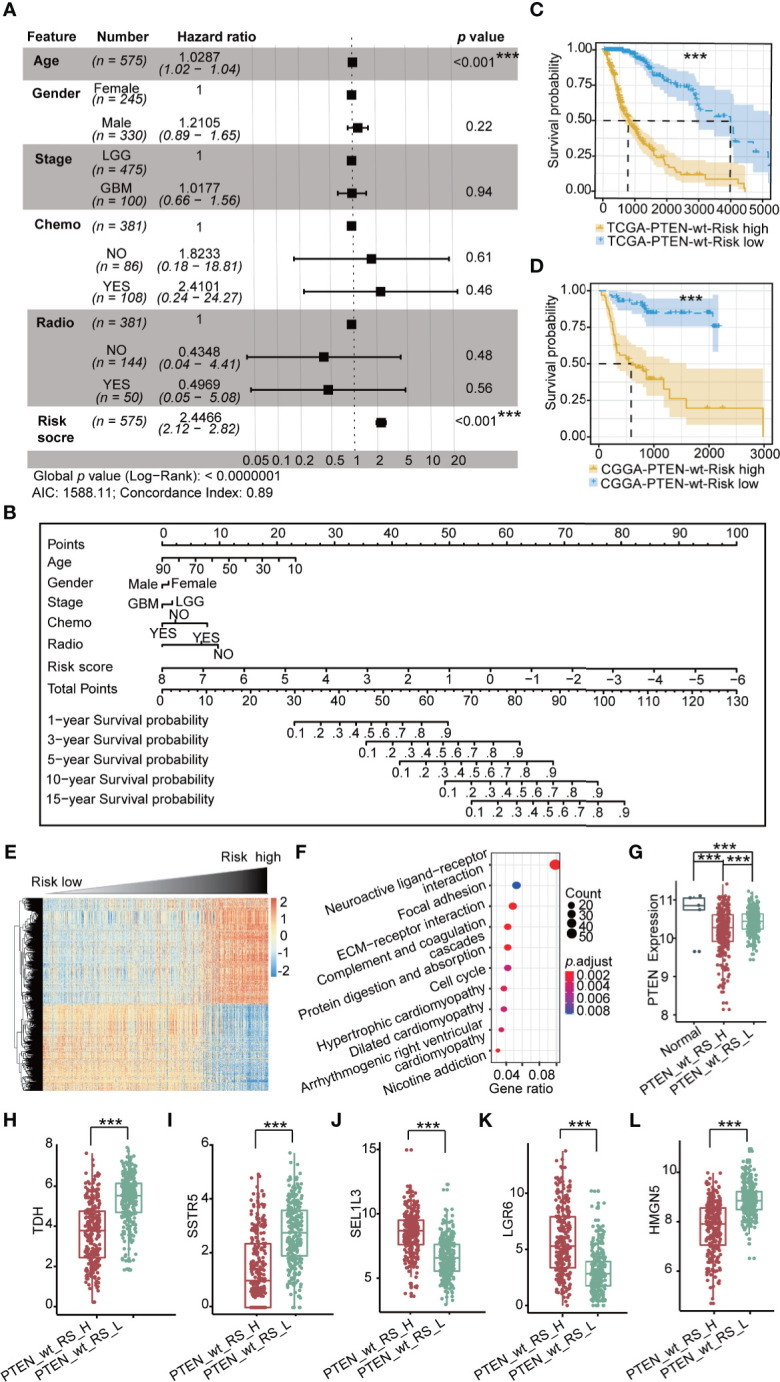
Prognostic risk model in TCGA PTEN-wt subgroup: **(A)** Multivariate Cox forest plot of risk score and clinical features in TCGA PTEN-wt subgroup (*n* = 575 , AIC = 1588.11, C-index = 0.89); **(B)** A nomogram incorporating risk score based on 44 OPR-DEGs (**Supplementary Table 5**, risk score in **Supplementary Table 1**) and clinical features in TCGA PTEN-wt subgroup; **(C)** Survival analysis (Kaplan Meier Curve) of high-risk score patients (PTEN_wt_RS_H, blue, *n* = 287) and low-risk score patients (PTEN_wt_RS_L, yellow, *n* = 287) in TCGA PTEN-wt subgroup (high-risk score and low-risk score separated from the median risk score of TCGA PTEN-wt subgroup); **(D)** Survival analysis of high-risk score patients (blue, *n* = 67) and low-risk score patients (yellow, *n* = 67) in CGGA PTEN-wt subgroup (high-risk score and low-risk score separated from the median risk score of CGGA PTEN-wt subgroup); **(E)** The heatmap of expression of DEGs (*n* = 1468, **Supplementary Table 6**) between PTEN_wt_RS_H and PTEN_wt_RS_L, and the risk score is ranked from low to high; **(F)** Pathway analysis of DEGs (*n* = 1468, **Supplementary Table 6**) between PTEN_wt_RS_H and PTEN_wt_RS_L; **(G)** Expression of *PTEN* in PTEN_wt_RS_H and PTEN_wt_RS_L; **(H–L)** Expression of *TDH*, *SSTR5*, *SEL1L3*, *LGR6*, *HMGN5* in PTEN_wt _RS_H and PTEN_wt _RS_L in TCGA. PTEN_wt_RS_H: high-risk score patients in TCGA PTEN-wt; PTEN_wt _RS_L: low-risk score patients in TCGA PTEN-wt; ***p < 0.001.

To establish the prognostic risk model of the PTEN-wt subgroup, log-rank test and univariate Cox analysis were performed using subgroup-specific DEGs (*n* = 147) of PTEN-wt to obtain PR-DEGs (*n* = 127, log-rank *p* < 0.05 and Wald test *p* < 0.05). Then, the PR-DEGs were used to fit L1-penalized (LASSO) Cox-pH regression model to obtain the OPR-DEGs (*n* = 44, **Supplementary Table 5**). The AUC value of the L1-penalized (LASSO) Cox-pH regression model was equal to 0.8807 (**Supplementary Figure 2A**), indicating the reliability of this model. We calculated the patients’ risk scores based on 44 OPR-DEGs according to the calculation formula cited in the materials and methods. To calculate the hazard of risk score, the risk score and clinical features of patients were analyzed by multivariate Cox analysis. The Concordance Index obtained by multivariate Cox analysis was 0.89, demonstrating the accuracy of our constructed risk score of polygenes and the prediction model in the PTEN-wt subgroup (**Figure 3A**). Notably, the multivariate Cox analysis showed the risk scores of patients were independent prognostic factors (*p* < 0.001), and the hazard ratio of patients with a high-risk score is 2.45-fold (**Figure 3A**). Finally, we calculated the nomogram by clinical features and the risk scores to predict clinical survival (**Figure 3B**). As shown in calibration plots by internal validation of Bootstrap Resamples (1000) of the TCGA-PTEN-wt dataset and external validation of the CGGA-PTEN-wt dataset (**Supplementary Figures 2B, C**), consistent results were observed between predicted and actual survival probability at 1-year, 3-year, and 5-year times (C-index of internal validation = 0.894, C-index of external validation = 0.808).

PTEN-wt subgroup in TCGA and CGGA dataset was separated into high-risk and low-risk patients by the median value of risk score. The Kaplan-Meier survival curve shows the prognosis of low-risk is significantly better than that of high-risk patients (**Figures 3C, D**; *p* < 0.001). We then screened DEGs between the high-risk patients and the low-risk patients and obtained 658 down-regulated, and 810 upregulated DEGs in the TCGA PTEN-wt subgroup (**Supplementary Figure 2D**, selection criteria: FDR < 0.05 and |log2FC| > 1, **Supplementary Table 6**). The patients’ risk score is ranked from low to high, and the DEGs between the high-risk patients and the low-risk patients are presented (**Figure 3E**, **Supplementary Table 6**). These upregulated DEGs in the high-risk patients were significantly enriched in 10 functionally KEGG signaling pathways, including neuroactive ligand-receptor interaction, focal adhesion, extracellular matrix (ECM)-receptor interaction, and cell cycle (**Figure 3F**, **Supplementary Table 6**). The clinical features of a high-risk and low-risk patient are shown in **Table 2**. We found the expression level of PTEN in high-risk patients was lower than low-risk patients in the PTEN-wt subgroup (*p* < 0.001), which showed that the risk score was also correlated with the level of PTEN expression (**Figure 3G**).

**Table 2:** Clinical information about risk high and risk low patients in PTEN-wt subgroups (*** significant difference).

Group	PTEN_wt Risk score high	PTEN_wt Risk score low	*p-value*
Patient(number)	287	288	
Age(median age)	53	39	<0.0001***
OS.time(median days)	480	765	<0.0001***
Male	60%(172)	55%(158)	
Female	40%(115)	45%(130)	
Chemotherapy	24%(70)	14%(4)	
Radiation therapy	10%(28)	3%(1)	
Astrocytoma	31%(90)	28%(81)	
Oligoastrocytoma	15%(43)	28%(81)	
Oligodendroglioma	20%(56)	43%(124)	
Glioblastoma	34%(98)	0.01%(2)	

OPR-DEGs, which best fit the patient’s survival time, are potential targets for driving glioma progression. The 44 OPR-DEGs in the TCGA PTEN-wt subgroup have 14 OPR-DEGs (Multivariate Cox: *p* < 0.05, **Supplementary Table 5**) that are independent prognostic genes and have a strong relationship with patient survival. Thus, 14/44 OPR-DEGs are optimal survival and risk prediction indicators in the TCGA PTEN-wt subgroup, for example, SSTR5 and TDH (**Supplementary Figure 2E**). Notably, 5/14 OPR-DEGs, including *TDH*, *SSTR5*, *HMGN5*, *LGR6*, and *SEL1L3*, also have different expressions between high-risk patients and low-risk patients in the TCGA PTEN-wt subgroup (**Figures 3H–L**, *p* < 0.001), suggesting that they are associated with the prognosis of PTEN-wt glioma.

### The Signature and Risk Score in the PTEN-mut Subgroup

**Figure 4:**
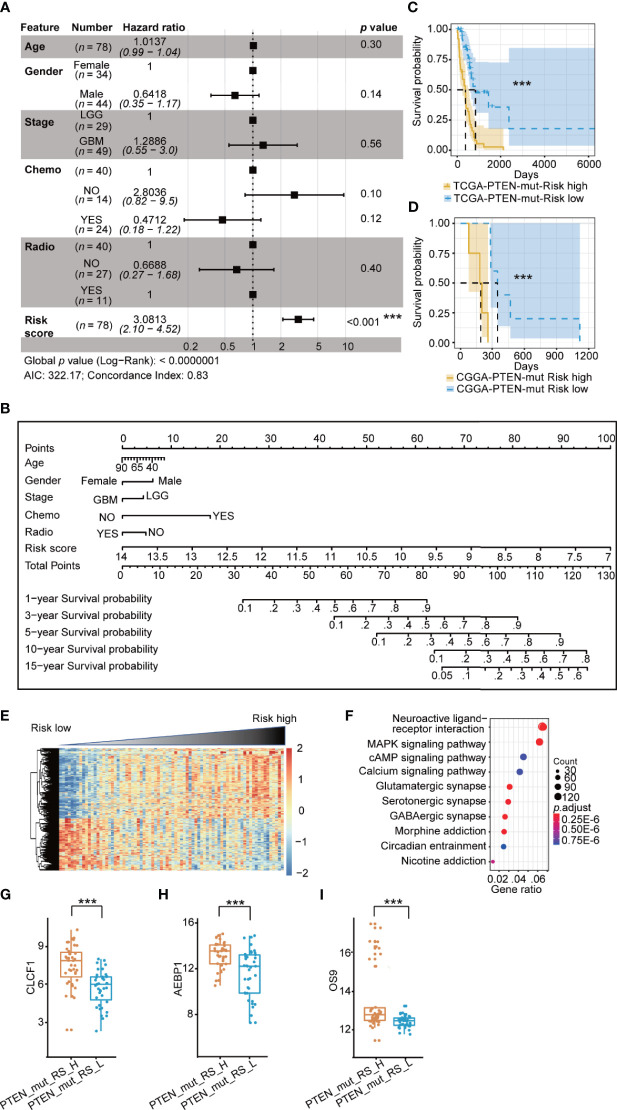
Prognostic risk model in TCGA PTEN-mut subgroup: **(A)** Multivariate Cox forest plot of risk score and clinical feature in TCGA PTEN-mut subgroup (*n* = 78 , AIC = 322.17, C-index = 0.83); **(B)** A nomogram incorporating risk score based on 11 OPR-DEGs (**Supplementary Table 7**, risk score in **Supplementary Table 1**) and clinical features in TCGA PTEN-mut subgroup; **(C)** Survival analysis (Kaplan Meier Curve) of high-risk score patients (PTEN_mut_RS_H, blue, *n* = 39) and low-risk score patients (PTEN_mut_RS_L, yellow, n = 39) in TCGA PTEN-mut subgroup (high-risk score and low-risk score separated from the median risk score of TCGA PTEN-mut subgroup); **(D)** Survival analysis of high-risk score patients (blue, *n* = 5) and low-risk score patients (yellow, *n* = 4) in CGGA PTEN-mut subgroup (high-risk score and low-risk score separated from the median risk score of CGGA PTEN-mut subgroup); **(E)** The heatmap of expression of DEGs (n = 476, **Supplementary Table 8**) between PTEN_mut_RS_H and PTEN_mut_RS_L, and the risk score is ranked from low to high; **(F)** Pathway analysis of DEGs (n = 476, **Supplementary Table 8**) between PTEN_mut_RS_H and PTEN_mut_RS_L; **(G–I)** Expression of *CLCF1* **(G)**, *AEBP1* **(H)** and *OS9* **(I)** in PTEN_mut _RS_H and PTEN_mut _RS_L. PTEN_mut_RS_H: high-risk score patients in TCGA PTEN-mut; PTEN_mut _RS_L: low-risk score patients in TCGA PTEN-mut; ***p < 0.001.

We used the same prognostic risk model in the PTEN-mut subgroup and obtained 11 OPR-DEGs (**Supplementary Table 7**). The AUC value was equal to 0.9414, indicating the model’s fine applicability (**Supplementary Figure 3A**). Notably, the multivariate Cox analysis showed that patients’ risk score in the PTEN-mut subgroup is an independent prognostic factor. The hazard ratio of patients with a high-risk score is 3.08 (**Figure 4A**). The 11 OPR-DEGs in the PTEN-mut subgroup have three independent OPR-DEGs (Multivariate Cox: *p* < 0.05), including *CLCF1*, *AEBP1*, and *OS9* (**Supplementary Table 7**). Meanwhile, the nomogram for the PTEN-mut subgroup was also constructed to predict individual patient survival rates (**Figure 4B**). The calibration plots by internal validation of Bootstrap Resamples (1000) in the TCGA-PTEN-mut subgroup demonstrate the high availability of predictive models (**Supplementary Figure 3B**, C-index of internal validation = 0.822). As the CGGA-PTEN-mut subgroup has only nine patients, external validation could not be performed.

As we predicted, the prognosis of low-risk patients was significantly better than that of high-risk patients (**Figures 4C, D**; *p* < 0.0001). The clinical features of the high-risk and low-risk patients are shown in **Table 3**. There are 273 upregulated and 203 down-regulated DEGs between the high-risk patients and the low-risk patients in the TCGA-PTEN-mut subgroup (**Supplementary Figure 3C**, selection criteria: FDR < 0.05 and |log2FC| > 1, **Supplementary Table 8**). The heatmap presents DEGs’ expression between the high-risk patients and the low-risk patients (**Figure 4E**, **Supplementary Table 6**). These upregulated DEGs in high-risk patients were significantly enriched in 5 KEGG signaling pathways, consisting of neuroactive ligand-receptor interaction, cytokine-cytokine receptor interaction, complement, and coagulation cascades, staphylococcus aureus infection, and nicotine addiction (**Figure 4F**, **Supplementary Table 8**). The expression of *CLCF1*, *AEBP1*, and *OS9* is significantly associated with the prognosis of PTEN-wt glioma, indeed which are significantly different between high-risk score patients and low-risk patients in the PTEN-mut subgroup (**Figures 4G–I**, *p* < 0.001).

**Table 3:** Clinical information about risk high and risk low patients in PTEN-mut subgroups (*** significant difference).

Group	PTEN_mut Risk score high	PTEN_mut Risk score low	*p-value*
Patient(number)	39	39	
Age(median age)	62	57	0.09
OS.time(median days)	360	535	<0.0001***
Male	67%(26)	46%(18)	
Female	33%(13)	54%(21)	
Chemotherapy	33%%(13)	14%(4)	
Radiation therapy	10%(4)	3%(1)	
Astrocytoma	15%(6)	33%(13)	
Oligoastrocytoma	0	10%%(4)	
Oligodendroglioma	3%(1)	13%(5)	
Glioblastoma	82%(32)	43%(17)	

### The Correlation Between Risk Score and Tumor Malignancy

To verify the correlation between risk score and glioma malignancy, we calculated ESTIMATEScore and TumorPurityScore for gliomas. Tumor microenvironment (TME) is closely related to the progression and prognosis of glioma (42–44). Studies have shown that TME significantly affects treatment response and clinical outcomes in cancer patients (45, 46). The components of TME are mainly resident stromal cells and immune cells, which are involved in the development of tumors (47–49). ESTIMATE (Estimation of STromal and Immune cells in MAlignant Tumor tissues using Expression data) is used for predicting tumor purity and the ratio of infiltrating stromal/immune cells (49). Higher ImmuneScore and StromalScore represent more immune/stromal components in TME. ESTIMATEScore, the sum of ImmuneScore and StromalScore, indicates the combined ratio of the immune and stromal components in TME (50). Ke-Wei et al. found that ESTIMATEScore was positively correlated with the survival rate at the tumor stage, suggesting that immune and stromal components were related to the invasion and metastasis of Lung adenocarcinoma (51). Glioma purity is highly correlated with major clinical and molecular characteristics, and low-purity glioma is more likely to be diagnosed as malignant. It is independently associated with reduced survival time (52). Thus, ESTIMATEScore and TumorPurityScore can bet the entry point to verify the malignant degree of glioma.

The ESTIMATE algorithm was applied for 653 glioma patients in TCGA. There was a high correlation between the risk score of patients and tumor purity score, ESTIMATEScore, StromalScore, and ImmuneScore (**Supplementary Figures 4A–D**). These patients with the high-risk score in the PTEN-mut subgroup have the highest ESTIMATEScore and lowest tumor purity score, which indicates the accuracy of the risk score based on OPR-DEGs in two subgroups. Patients in each subgroup were visually represented linearly (**Supplementary Figure 4E**). The worst survival patients with the high-risk score in the PTEN-mut subgroup were the most aggressive GBM, mostly older than 60, entirely had high ESTIMATEScore, and altogether had died at the last follow-up. On the contrary, the best survival patients with low-risk in the PTEN-wt subgroup belong to the low-grade glioma; their ages were between 20 and 60; most patients had low ESTIMATEScore, and were still alive until the last follow-up. These results demonstrate consistency between risk score and clinical survival rate.

### Validation of Risk Score and Prognostic Genes in Clinical Significance

To verify the clinical value of our newly established risk model and prognostic genes, we firstly calculated and found that risk scores were higher in the PTEN-mut than in the PTEN-wt subgroup both in GBM and LGG patients (**Supplementary Figure 5A**). We used PCA (Principal Component Analysis) to convert the dimensionality reduction of all genes into three-dimensional coordinates. Surprisingly, a similar pattern was obtained from PCA analysis by using all genes or the prognostic genes from indicated glioma groups, suggesting prognostic genes identified by our risk model reflect the variation among different types of patients (**Supplementary Tables 5**, **7** and **Supplementary Figure 5B**).

**Figure 5:**
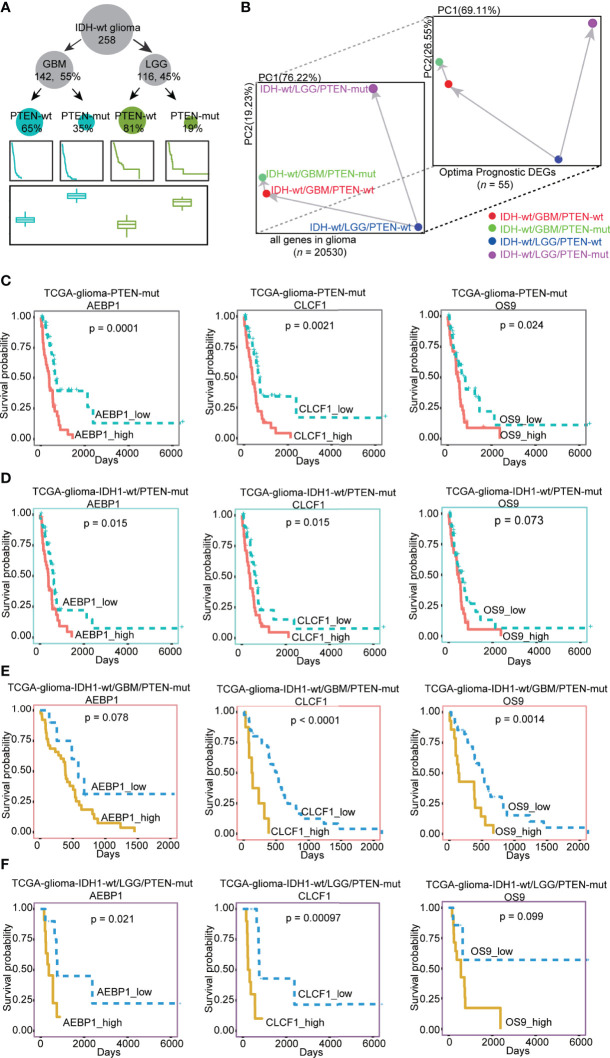
**(A)** The number of patients, survival rates, and the risk score established by our model in different types of IDH-wt glioma dataset. There are 258 IDH-wt glioma patients, 142 (55%) IDH-wt/GBM, 116 (45%) IDH-wt/LGG, 93 (65%) IDH-wt/GBM/PTEN-wt, 49 (35%) IDH-wt/GBM/PTEN-mut, 94 (81%) IDH-wt/LGG/PTEN-wt and 22 (19%) IDH-wt/LGG/PTEN-mut; **(B)** PCA plots of different type of IDH-wt glioma (IDH-wt/GBM/PTEN-wt, IDH-wt/GBM/PTEN-mut, IDH-wt/LGG/PTEN-wt and IDH-wt/LGG/PTEN-mut) by the expression of all genes and optimal prognostic DEGs (OPR-DEGs of PTEN-wt and PTEN-mut in **Supplementary Tables 5** and **7**, *n* = 55); **(C–F)** Survival analysis of the expression level of AEBP1, CLCF1 and OS9 in different types of glioma with PTEN-mut, high expression and low expression of these three genes in TCGA PTEN-mut and TCGA IDH-wt/PTEN-mut were grouped by median values of expression ( **C**: AEBP1_high: n = 39, AEBP1_low: n = 39, CLCF1_high: *n* = 39, CLCF1_low: *n* = 39, OS9_high: *n* = 39; OS9_low: *n* = 39 in TCGA PTEN-mut; D: AEBP1_high: *n* = 35, AEBP1_low: *n* = 35, CLCF1_high: *n* = 35, CLCF1_low: *n* = 35, OS9_high: *n* = 35; OS9_low: *n* = 35 in IDH-wt/PTEN-mut), in IDH-wt/GBM/PTEN-mut and IDH-wt/LGG/PTEN-mut grouped by the best dividing points form *tinyarray* package of R ( **(E)**: AEBP1_high: n = 39, AEBP1_low: n = 10, CLCF1_high: n = 8, CLCF1_low: n = 41, OS9_high: n = 14; OS9_low: n = 35 in IDH-wt/GBM/PTEN-mut; **F**: AEBP1_high: n = 11, AEBP1_low: n = 11, CLCF1_high: n = 10, CLCF1_low: n = 12, OS9_high: n = 14; OS9_low: n = 8 in IDH-wt/LGG/PTEN-mut).

Secondly, WHO identified IDH1 mutation as an essential glioma classification criteria in 2016 (7). We divided the TCGA-glioma dataset into IDH wild-type (IDH-wt) and mutant types (IDH-mut). Only 2% of patients in the IDH-mut group had PTEN mutations (*n* = 7), compared with 28% in IDH-wt (*n* = 71, **Supplementary Figure 5C**). We then focused on the patients with IDH-wt and showed that PTEN-mut had higher risk scores than PTEN-wt groups (**Supplementary Figure 5C**). PCA analysis showed prognostic genes identified by our risk model represent the variation of IDH -wt patients with different PTEN status, strongly indicating the accuracy of risk model (**Supplementary Tables 5**, **7** and **Supplementary Figure 5D**). We then concentrate on glioblastoma only or lower grades only to analyze the effect of PTEN states in IDH-wt, including 65% IDH-wt/GBM/PTEN-wt, 35% IDH-wt/GBM/PTEN-mut, 81% IDH-wt/LGG/PTEN-wt, and 19% IDH-wt/LGG/PTEN-mut (**Figure 5A**). Consistently, the risk score of PTEN-mut was higher than that of PTEN-wt in both IDH_wt/GBM and IDH_wt/LGG patients (**Figure 5A**). PCA results also supported the idea that the risk model plays a role in clinical significance evidenced by a similar pattern shown in more defined types of glioma (**Supplementary Tables 5**, **7** and **Figure 5B**). To verify that previous identified prognostic genes of TCGA-glioma PTEN-mut are also the prognostic genes in IDH1-wt patients with PTEN-mut, we observed that AEBP1, CLCF1, and OS9 were significantly prognostic genes in IDH1-wt, IDH1-wt/GBM, or IDH-wt/LGG gliomas with PTEN-mut (**Figures 5C–F**). Altogether, these data demonstrate the reliability of the risk model and the potential therapeutic targets of the three genes in PTEN-mut glioma for clinical use of IDH-wt patients.

### Targeting *CLCF1*, *AEBP1*, and *OS9* Genes in PTEN-mut Glioma to Explore the Mechanism and Potential Treatment

Due to the higher malignancy of the PTEN mutant glioma, we focused on the mechanism and treatment of the PTEN-mut subgroup. Subgroup-specific *CLCF1*, *AEBP1*, and *OS9* are the independent OPR-DEGs and the differential expression genes between high-risk patients and low-risk patients in the PTEN-mut subgroup (**Figures 4G–I** and **Supplementary Table 7**). Thus, they are associated with tumor progression and may be critical prognostic targets for gliomas with mutant *PTEN*, and more precisely in patients with IDH1-wt/PTEN-mut (**Figures 5C–F**). To explore the underlying mechanism of PTEN-mut glioma progression, the subgroup-specific and independent indicators, *CLCF1*, *AEBP1*, and *OS9*, are analyzed for PPI (Protein-Protein Interactions, **Supplementary Figures 6A–C**). In the PPI network of *CLCF1* (**Supplementary Figure 6A**), the signal transducer and activator of transcription 3 (*STAT3*) is upregulated in PTEN-mut but not in the PTEN-wt subgroup (PTEN-mut *vs.* Normal, PTEN-wt *vs.* Normal), showing *STAT3* is correlated with PTEN-mut glioma. Similarly, in the PPI network of *AEBP1* (**Supplementary Figure 6B**), ATP/GTP binding protein 1 (*AGTPBP1*), tumor protein P53 target 5 (*TP53TG5*), leucine-rich repeat transmembrane neuronal 4 (*LRRTM4*), transcription factor 3 (*TCF3*) and aspartoacylase (*ASPA*) are DEGs between PTEN-mut subgroup and Normal patients and may be associated with poor prognosis of PTEN-mut subgroup. In the PPI network of *OS9* (**Supplementary Figure 6C**), *OS9* and derlin 2 (*DERL2*) involve in protein processing in the endoplasmic reticulum and complicate CFTR (Cystic fibrosis transmembrane conductance regulator) causes cystic fibrosis pathways, which may be associated with the prognosis of glioma with mutant *PTEN*.

**Figure 6:**
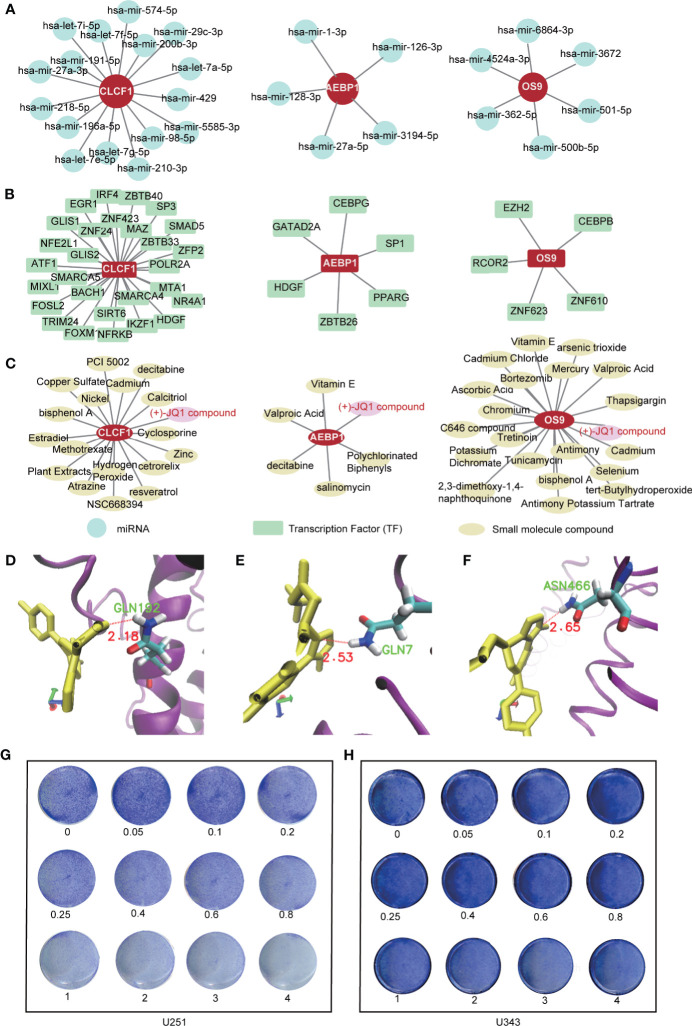
Targeted treatment for PTEN-mut glioma: **(A)** The miRNA-gene network of *CLCF1*, *AEBP1* and *OS9* by NetworkAnalyst (Materials and methods 2.6); **(B)** The TFs-gene network of CLCF1, AEBP1 and OS9 by NetworkAnalyst (Materials and methods 2.6); **(C)** The protein-chemical network of CLCF1, AEBP1, and OS9 by NetworkAnalyst (Materials and methods 2.6); **(D–F)** The docking site between CLCF1, AEBP1, OS9 with (+)-JQ1 by Autodock (Materials and methods 2.7); **(G)** Colony formation assay was performed in U251(PTEN- deficiency) cells treated by (+)-JQ1 with different concentration (Materials and methods 2.8); **(H)** Colony formation assay was performed in U343 cells treated by (+)-JQ1 with different concentration (*Materials and Methods 2.8*).

For the targeted treatment of PTEN-mut glioma, we constructed the miRNA-gene (**Figure 6A**), TFs-genes (**Figure 6B**), and protein-chemical network (**Figure 6C**). *CLCF1*, *AEBP1*, and *OS9* have different binding miRNA and TFs, respectively. Notably, the small compound (+)-JQ1 can bind to all these three proteins (CLCF1, AEBP1, and OS9). To verify that (+)-JQ1 binds with CLCF1, AEBP1, and OS9, we predicted the structure of CLCF1, AEBP1, and OS9, and docked the binding sites of (+)-JQ1 with these three proteins. Docking results showed that (+)-JQ1 binds to all three proteins stably (**Figures 6D–F**). Furthermore, we treated U251(PTEN-deficient) and U343 (PTEN-wt) cells with different concentrations of (+)-JQ1 (**Figures 6G, H**). Results showed that U251 cells were more sensitive to (+)-JQ1. This experimental data indicated that (+)-JQ1 indeed had a potential medicinal effect on the PTEN-mut subgroup. Therefore, (+)-JQ1 potentially competes for and binds to the CLCF1, AEBP1, and OS9 proteins, resulting in the interruption of the pro-oncogenic pathway in PTNE-mut gliomas. These results highlight the therapeutic potential of (+)-JQ1 in treating PTEN-mut gliomas and demand extensive *in vitro* and *in vivo* studies for experimental verifications.

## Discussion

Gliomas are the most lethal primary brain tumors lacking effective treatment, and the *PTEN* mutation event significantly reduces the survival rates of glioma patients. Depending on the *PTEN* status, glioma can be divided into two apparent subgroups: the PTEN-wt and PTEN-mut. In this study, we established prognostic risk models for the two subgroups, respectively. In summary, we first analyzed the expression of subgroup-specific tumor DEGs and the enriched pathways. Based on the subgroup-specific DEGs, we successfully screened subgroup-specific optimal prognostic signature (OPR-DEGs) and calculated risk scores for individual patients based on their OPR-DEGs. *CLCF1*, *AEPB1*, and *OS9* in the PTEN-mut subgroup are the optimal and independent prognostic signatures and can be used as potential targets for the diagnosis, prediction, and treatment of *PTEN*-mut glioma. Finally, we found miRNAs and TFs that interacted with *CLCF1*, *AEPB1*, and *OS9* genes and chemicals that interacted with CLCF1, AEPB1, and OS9 proteins. We showed the therapeutic potential of the gene or protein interacting agents, especially (+)-JQ1.

Mutational loss of PTEN is an established malignancy event and PTEN significantly influences therapeutic efficacy in glioma (23, 25, 27, 28). PTEN status of glioma patients according to their unique signatures improve the survival rate. Chen et al. reported symbiotic macrophage-glioma cell interactions to cause synthetic lethality in PTEN-Null glioma (53). In 2006, Parsa et al. had shown tumor-specific T cells more effectively target human glioma expressing wild-type PTEN than those expressing mutant PTEN (54). Conversely, we demonstrated that the oncogenic role of Smurf1 promotes GBM growth by mediating PTEN ubiquitylation and degradation (55). Additionally, we also realized that PTEN status is a prognostic marker in all grades of gliomas and LGG only, but not in the GBM group only. These may due to PTEN defect is an initial cause of malignancy in glioma patients. The progression of GBM leads to acquired secondary mutations (6). In this rationale, we combined the RNA-seq data of GBM and LGG to build a risk model. To verify the clinical significance of our risk model, we focused on IDH-wt glioma first, then concentrate on GBM only or LGG only. We identified that PTEN status is also a critical prognostic factor in terms of clinical classification of patients, such as in IDH1-wt, IDH1-wt/GBM, and IDH1-wt/LGG (**Figure 5**).

In the PTEN-wt subgroup, 44 OPR-DEGs were fitted with the best survival time. Among the 44 OPR-DEG genes, 14 genes were independent prognostic signatures (Multivariable Cox, *p* < 0.05, **Supplementary Table 5**). *SSTR5* has the cytostatic effects of somatostatin in C6 glioma cells by activating PTPeta (protein tyrosine phosphatase eta) and inhibiting extracellular signal-regulated kinase (ERK)1/2 activity (56). *HMGN5*, as a potential oncogene, is highly expressed in breast cancer and hormone-induced mouse uterine adenocarcinoma. Qu et al. found suppression of HMGN5 caused a delay in the growth of human glioma cells (57). *MEGF10*, a critical IDH mutation predictor, plays an important role in cell migration, apoptosis, and proliferation (58). *SSTR5*, *HMGN5*, and *MEGF10* have been associated with glioma, but they are not associated with PTEN status. *HSPC159* induces epithelial-mesenchymal transition, activates the PI3K/protein kinase B (Akt) pathway, and promotes proliferation and metastasis in breast cancer (59). Studies reported that upregulated SLC6A6 induces tumorigenesis and reduces clinical outcomes in gastric cancer (60). Pei et al. found that loss of LAMC2 changes epithelial-mesenchymal transition and inhibits angiogenesis in cholangiocarcinoma *via* inactivation of the epidermal growth factor receptor (EGFR) signaling pathway (61). LGR6, as a tumor suppressor, belongs to the rhodopsin-like seven-transmembrane domain receptor superfamily and is a high-affinity receptor of R-spondins with potential functions (62). These genes have been reported as markers for other tumors but have not been studied in gliomas and may be potential targets for gliomas, especially the PTEN-wt subgroup.

In the PTEN-mut subgroup, 11 OPR-DEGs were fitted with the best survival time. Out of 11, three OPR-DEGs (*CLCF1*, *AEBP1*, and *OS9)* are independent prognostic signatures and are upregulated in PTEN-mut high-risk patients (**Figures 4G–I** and **Supplementary Table 7**). *CLCF1* is a neurotrophic and B cell-stimulating factor belonging to the interleukin-6 (IL-6) family. Studies have found that *CLCF1* induced phosphorylation of signal transducer and activator of transcription 3 (*STAT3*) (**Supplementary Figure 6D**) (63). *STAT3* is upregulated in PTEN-mut but not in the PTEN-wt subgroup (PTEN-mut *vs.* Normal, PTEN-wt *vs.* Normal), showing *STAT3* is correlated with PTEN-mut glioma (**Supplementary Figure 6A**). Notably, *STAT3* participates in three signaling pathways, including IL-6 family signaling, Janus kinase (JAK)/STAT signaling pathway, and EGFR/RTK pathway. Continuous activation of *STAT3* in GBM with PTEN loss can induce cell proliferation, anti-apoptosis, maintenance of glioma stem cells, tumor invasion, angiogenesis, and immune evasion (64, 65). Inhibition of *CLCF1* to reduce phosphorylation of *STAT3* may be an effective strategy for treating glioma with mutant PTEN. *AEBP1* is involved in the regulation of adipogenesis, mammary gland development, inflammation, macrophage cholesterol homeostasis, and atherogenesis in various human tumors (66–70). Swati Sinha et al. reported that the downregulation of *AEBP1* in PTEN-deficient cells activated cell death through a caspase-independent pathway that is different from PTEN-wt glioma (**Supplementary Figure 6E**) (66, 67). Our study further confirms the importance and potential therapeutic intervention implications of *CLCF1* and *AEBP1* in *PTEN* mutant glioma. We also predicted that *OS9* is the best independent prognostic signature of the PTEN-mut subgroup. The protein encoded by *OS9* is highly expressed in osteosarcoma and binds to hypoxia-inducible factor 1 (*HIF-1*), a key regulator of hypoxia response and angiogenesis (71–73). Studies on *OS9* in glioma have not been reported, but our model demonstrated the strong correlation between *OS9* and survival time, indicating that *OS9* is also a potential target for glioma. We analyzed the gene-miRNA, gene-TF, and protein-chemical interaction networks to guide the targeted therapy (**Figures 6A-C**). (+)-JQ1 compound is a triazole-diazepine compound family member, which functions as a pan-BET (bromodomain and extra-terminal motif) family inhibitor (74). (+)-JQ1 is known to suppress cell proliferation and can be used as a therapeutic drug for many cancers, including multiple myeloma and acute myeloid leukemia (75). Following a single intraperitoneal dose of (+)-JQ1 (50 mg/kg) in male mice, Matzuk et al. measured the (+)-JQ1 concentration in serum, testis, and brain. They observed nearly uniform blood-brain barrier permeability after single-dose pharmacokinetic studies (AUCbrain/AUCplasma = 98%) (76). Korb et al. in 2015 examined whether JQ1 affects brain function in mice. They injected wild-type adult male mice with (+)-JQ1 (50 mg/kg) daily for 1 week or 3 weeks before performing behavioral tests, and the result proved that (+)-JQ1 has excellent blood-brain barrier permeability (77). (+)-JQ1 compound may combine and inhibit the CLCF1, AEBP1, and OS9 interacting proteins, and hence, it can be a potential treatment option for PTEN-mut gliomas.

## Conclusion

In conclusion, we established a prognostic risk model and risk score in glioma with different PTEN status and obtained 14 independent prognostic signatures in PTEN-wt glioma and 3 independent prognostic signatures in PTEN-mut glioma. Thus, in the PTEN-mut glioma, *CLCF1*, *AEBP1*, and *OS9*, which are significantly associated with survival time, may induce glioma progression and are critical targets for diagnosis, prognosis prediction, and treatment, giving therapeutic recommendations to glioma with mutant PTEN.

## Data Availability Statement

The original contributions presented in the study are included in the article/**Supplementary Material**. Further inquiries can be directed to the corresponding authors.

## Author Contributions

PZ contributed to the conception, design, and drafting of the manuscript. QX provided useful comments and suggestions. LD and QX revised the manuscript. All authors contributed to the article and approved the submitted version.

## Funding

This work was supported by grants from the Beijing Natural Science Foundation (Z190018), the National Natural Science Foundation of China (81870123), the China Postdoctoral Science Foundation Grant (2018M641206), the National Science Foundation for Young Scientists of China (81902545), Beijing Institute of Technology Research Fund Program for Young Scholars (XSQD-202110002).

## Conflict of Interest

Authors LD, PZ and QX applied for a patent in China, the patent application number is 202010977490.6.

The remaining authors declare that the research was conducted in the absence of any commercial or financial relationships that could be construed as a potential conflict of interest.

## Supplementary Material

Supplementary Figure 1
**(A)** Survival analysis of TP53-wt (blue, *n* = 466) and TP53-mut (yellow, *n* = 430) in TCGA dataset; **(B)** Survival analysis of IDH1-wt (blue, *n* = 481) and IDH1-mut (yellow, *n* = 415) in TCGA dataset; **(C)** Survival analysis of TTN-wt (blue, *n* = 531) and TTN-mut (yellow, *n* = 365) in TCGA dataset; **(D)** Survival analysis of ATRX-wt (blue, *n* = 630) and ATRX-mut (yellow, *n* = 266) in TCGA dataset; **(E)** Survival analysis of PTEN-wt (pink, *n* = 733), patients with unreported *PTEN* mutant sites (yellow, *n* = 70) and patients with reported *PTEN* mutant sites (blue, *n* = 88) in TCGA dataset.

Supplementary Figure 2
**(A)** ROC curve of L1-Penalized (LASSO) Cox-pH regression model in TCGA PTEN-wt subgroup; **(B)** The calibration plots by internal validation of Bootstrap Resamples (1000) in TCGA-PTEN-wt dataset; **(C)** The calibration plots by external validation of CGGA PTEN-wt dataset (*n* = 135); **(D)** The volcano plot of DEGs between high-risk patients and low-risk patients in TCGA PTEN-wt subgroup (DGEs: n = 1468); **(E)** The decrease expression of SSTR5 and TDH results in a poor prognosis in PTEN-wt patients.

Supplementary Figure 3
**(A)** ROC curve of L1-Penalized (LASSO) Cox-pH regression model in TCGA PTEN-mut subgroup; **(B)** The calibration plots by internal validation of Bootstrap Resamples (1000) in TCGA-PTEN-mut dataset; **(C)** The volcano plot of DEGs between high-risk score patients and low-risk score patients in TCGA PTEN-mut subgroup (DGEs: n = 476).

Supplementary Figure 4 The correlation between risk score with malignancy of glioma: **(A–D)** TumorPurityScore **(A)**, ESTIMATEScore **(B)**, StromalScore **(C)**, and ImmuneScore **(D)** in patients with a different risk score, including high-risk score patients in TCGA PTEN-mut subgroup (PTEN_mut_RS_H), low-risk score patients in TCGA PTEN-mut subgroup (PTEN_mut_RS_H), high-risk score patients in TCGA PTEN-wt subgroup (PTEN_wt _RS_H), and low-risk score patients in TCGA PTEN-mut subgroup (PTEN_wt _RS_L); **(E)** The alluvial diagram of TCGA glioma dataset, each line represents a patient.

Supplementary Figure 5
**(A)** The number of patients, survival rates, and the risk score established by our model in different types of TCGA glioma dataset; **(B)** PCA plots of different type of glioma by the expression of all genes and Optimal Prognostic DEGs (OPR-DEGs in PTEN-wt and PTEN-mut); **(C)** The number of patients, survival rates, and the risk score established by our model in IDH-wt glioma; **(D)** PCA plots of different type of IDH-wt glioma by the expression of all genes and Optimal Prognostic DEGs (OPR-DEGs in PTEN-wt and PTEN-mut, **Supplementary Tables 5** and **7**).

Supplementary Figure 6
**(A–C)** The protein-protein (PPI) network of CLCF1 **(A)**, AEBP1 **(B)**, and OS9 **(C)**; **(D)** The mechanism of cell proliferation induced by CLCF1; **(E)** The mechanism of cell death induced by AEBP1 downregulation.

Supplementary Table 1 Clinical features and normalized count of glioma patients inTCGA and CGGA.

Supplementary Table 2 PTEN mutation list by varscan2 in TCGA glioma patients (*Materials and Methods 2.1*).

Supplementary Table 3 PTEN-wt subgroup-specific DEGs.

Supplementary Table 4 PTEN-mut subgroup-specific DEGs.

Supplementary Table 5 44 OPR-DEGs in the PTEN-wt subgroup.

Supplementary Table 6 DEGs between high risk patients and low risk patients in TCGA PTEN-wt subgroup.

Supplementary Table 7 11 OPR-DEGs in the PTEN-mut subgroup.

Supplementary Table 8 DEGs between high risk patients and low risk patients in TCGA PTEN-mut subgroup.

## Glossary

AEBP1: adipocyte enhancer-binding protein 1
AGTPBP1: ATP/GTP binding protein 1
AKT: protein kinase B
ASPA: aspartoacylase
AUC: area under the receiver operating characteristic curve
CENPV: centromere protein V
CFTR: cystic fibrosis transmembrane conductance regulator
CGGA: The Chinese Glioma Genome Atlas
CLCF1: cardiotrophin like cytokine factor 1
CVL: cross-validation likelihood
DEGs: differentially expressed genes
DEGs-all: DEGs from TCGA glioma vs. Normal
DEGs-mut: DEGs from TCGA PTEN-wt vs. Normal
DEGs-wt: DEGs from TCGA PTEN-wt vs. Normal
DERL2: derlin 2
ECM: extracellular matrix
EGFR: epidermal growth factor receptor
ERCC1: ERCC excision repair 1
ERK: extracellular signal-regulated kinase
EWSAT1: ewing sarcoma associated transcript 1
FDR: fold discovery rate
FGF-2: fibroblast growth factor 2
FGFR2: fibroblast growth factor receptor 2
GBM: glioblastoma multiforme
GSEA: Gene set enrichment analysis
HDAC7: histone deacetylase 7
HDGF: heparin-binding growth factor
HGG: high-grade glioma
HIF-1: hypoxia-inducible factor 1
HMGN5: high mobility group nucleosome binding domain 5
IDH: Isocitrate dehydrogenase
IGF-1R: insulin-like growth factor 1 receptor
IL: interleukin
JAK: Janus kinase
KEGG: Kyoto Encyclopedia of Genes and Genomes
LAMC2: laminin subunit gamma 2
LGG: low-grade glioma
LGR6: leucine rich repeat containing G protein-coupled receptor 6
LRRTM4: leucine rich repeat transmembrane neuronal 4
MAPK: mitogen-activated protein kinase
MEGF10: multiple epidermal growth factor like domains 10
MGMT: O6-methylguanine-DNA methyltransferase
miRNA: microRNA
NF-κB: Nuclear factor kappa B
OPR-DEGs: Optimal PR-DEGs
OS9: OS9 endoplasmic reticulum lectin
PCA: Principal Component Analysis
PD-1: Programmed cell death 1
PI3K: phosphatidylinositol-3-kinase
PPI: protein-protein interaction
PR-DEGs: Prognostic DEGs
PTEN: phosphatase and tensin homologous gene
PTEN-wt: glioma with wild type *PTEN* subgroup
PTEN-mut: glioma with mutant *PTEN* subgroup
SEL1L3: SEL1L family member 3
SLC6A6: solute carrier family 6 member 6
SSTR5: somatostatin receptor 5
STAT: signal transducer and activator of transcription
STAT3: signal transducer and activator of transcription 3
TCF3: transcription factor 3
TCGA: The Cancer Genome Atlas Program
TDH: L-Threonine Dehydrogenase
TF: transcriptional factor
TM4SF20: transmembrane 4 L six family member 20
TP53TG5: tumor protein P53 target 5
TWIST: twist-related protein
WHO: the World Health Organization
VEGF: vascular endothelial growth factor
ZNF326: zinc finger protein 326

## References

[1] Kleihues. World Health Organization Classification of Tumors. Cancer (2000) 88:2887. 10.1002/1097-0142(20000615)88:12<2887::AID-CNCR32>3.0.CO;2-F 10870076

[2] van den Bent MJ. Interobserver Variation of the Histopathological Diagnosis in Clinical Trials on Glioma: A Clinician’s Perspective. Acta Neuropathol (2010) 120:297–304. 10.1007/s00401-010-0725-7 20644945 PMC2910894

[3] See SJ Gilbert MR. Anaplastic Astrocytoma: Diagnosis, Prognosis, and Management. Semin Oncol (2004) 31:618–34. 10.1053/j.seminoncol.2004.07.004 15497115

[4] Butowski NA Sneed PK Chang SM. Diagnosis and Treatment of Recurrent High-Grade Astrocytom. J Clin Oncol (2006) 24:1273–80. 10.1200/JCO.2005.04.7522 16525182

[5] Weller M Sneed W Aldape K Brada M Berger M Pfister SM. Glioma. Nat Rev Dis Primers (2015) 1(1):15040. 10.1038/nrdp.2015.40 27188790

[6] Ceccarelli M Barthel Floris P Malta Tathiane M Sabedot Thais S Salama Sofie R Murray Bradley A. Molecular Profiling Reveals Biologically Discrete Subsets and Pathways of Progression in Diffuse Gliom. Cell (2016) 164:550–63. 0.1016/j.cell.2015.12.028 10.1016/j.cell.2015.12.028 PMC4754110 26824661

[7] Louis DN Perry A Reifenberger G Von Deimling A Figarellabranger D Cavenee WK. The 2016 World Health Organization Classification of Tumors of the Central Nervous System: A Summary. Acta Neuropathologica (2016) 131:803–20. 10.1007/s00401-016-1545-1 27157931

[8] Ichimura K Pearson DM Kocialkowski S Backlund LM Chan R Jones DT. IDH1 Mutations Are Present in the Majority of Common Adult Gliomas But Rare in Primary Glioblastomas. Neuro Oncol (2009) 11:341–7. 10.1215/15228517-2009-025 PMC2743214 19435942

[9] Watanabe T Nobusawa S Kleihues P Ohgaki H. IDH1 Mutations are Early Events in the Development of Astrocytomas and Oligodendrogliomas. Am J Pathol (2009) 174:1149–53. 10.2353/ajpath.2009.080958 PMC2671348 19246647

[10] Nobusawa S Watanabe T Kleihues P Ohgaki H. IDH1 Mutations as Molecular Signature and Predictive Factor of Secondary Glioblastomas. Clin Cancer Res (2009) 15:6002–7. 10.1158/1078-0432.CCR-09-0715 19755387

[11] Yan H Parsons DW Jin G Mclendon RE Rasheed BKA Yuan W. IDH1 and IDH2 Mutations in Glioma. New Engl J Med (2009) 360:765–73. 10.1016/S0513-5117(09)79085-4 PMC2820383 19228619

[12] Turcan S Rohle D Goenka A Walsh LA Fang F Yilmaz E. IDH1 Mutation Is Sufficient to Establish the Glioma Hypermethylator Phenotype. Nature (2012) 483:479–83. 10.1038/nature10866 PMC3351699 22343889

[13] Songtao Q Lei Y Si G Yanqing D Huixia H Xuelin Z. IDH Mutations Predict Longer Survival and Response to Temozolomide in Secondary Glioblastoma. Cancer Sci (2012) 103:269–73. 10.1111/j.1349-7006.2011.02134.x 22034964

[14] Mondesir J Willekens C Touat M de Botton S. IDH1 and IDH2 Mutations as Novel Therapeutic Targets: Current Perspectives. J Blood Med (2016) 7:171–80. 10.2147/JBM.S70716 PMC5015873 27621679

[15] Hegi ME Diserens A Gorlia T Hamou M De Tribolet N Weller M. MGMT Gene Silencing and Benefit From Temozolomide in Glioblastom. New Engl J Med (2005) 352:997–1003. 10.1056/NEJMoa043331 15758010

[16] Villa C Miquel C Mosses D Bernier M Di Stefano AL. The 2016 World Health Organization Classification of Tumours of the Central Nervous System. Presse Med (2018) 47(11-12 Pt 2):e187–e200. 10.1007/s00401-016-1545-1 30449638

[17] Cancer Genome Atlas Research N Brat DJ Verhaak RG Aldape KD Yung WK Salama SR. Comprehensive, Integrative Genomic Analysis of Diffuse Lower-Grade Glioma. N Engl J Med (2015) 372:2481–98. 10.1056/NEJMoa1402121 PMC4530011 26061751

[18] Phillips HS Kharbanda HS Chen S Forrest R Soriano WF Wu RH. Molecular Subclasses of High-Grade Glioma Predict Prognosis, Delineate a Pattern of Disease Progression, and Resemble Stages in Neurogenesis. Cancer Cell (2006) 9:157–73. 10.1016/j.ccr.2006.02.019 16530701

[19] Zhang P Xia Q Liu L Li S Dong L. Current Opinion on Molecular Characterization for GBM Classification in Guiding Clinical Diagnosis, Prognosis, and Therapy. Front Mol Biosci (2020) 7. 10.3389/fmolb.2020.562798 PMC7506064 33102518

[20] Oldrini B Curiel–Garcia A Marques C Matia V Uluckan O Grana–Castro O. Somatic Genome Editing With the RCAS-TVA-CRISPR-Cas9 System for Precision Tumor Modeling. Nat Commun (2018) 9:1466. 10.1038/s41467-018-03731-w 29654229 PMC5899147

[21] Cancer Genome Atlas Research, N. Comprehensive Genomic Characterization Defines Human Glioblastoma Genes and Core Pathways. Nature (2008) 455:1061–8. 10.1038/nature07385 PMC2671642 18772890

[22] Verhaak RG Hoadley KA Purdom E Wang V Qi Y Wilkerson MD. Integrated Genomic Analysis Identifies Clinically Relevant Subtypes of Glioblastoma Characterized by Abnormalities in PDGFRA, IDH1, EGFR, and NF1. Cancer Cell (2010) 17:98–110. 10.1016/j.ccr.2009.12.020 20129251 PMC2818769

[23] Fults D. Immunocytochemical Mapping of the Phosphatase and Tensin Homolog (PTEN/MMAC1) Tumor Suppressor Protein in Human Gliomas. Neuro-Oncology (2000) 2:71–9. 10.1215/15228517-2-2-71 PMC1919512 11303623

[24] Yang H Han F Hu R Liu J Sui J Xiang X. PTEN Gene Mutations Correlate to Poor Prognosis in Glioma Patients: A Meta-Analysis. OncoTargets Ther (2016) 3485. 10.2147/OTT.S99942 PMC4913532 27366085

[25] Zhao J Chen AX Gartrell RD Silverman AM Aparicio L Chu T. Immune and Genomic Correlates of Response to Anti-PD-1 Immunotherapy in Glioblastoma. Nat Med (2019) 25:462–9. 10.1038/s41591-019-0349-y PMC6810613 30742119

[26] Kessler T Sahm F Blaes J Osswald M Rübmann P Milford D. Glioma Cell VEGFR-2 Confers Resistance to Chemotherapeutic and Antiangiogenic Treatments in PTEN-Deficient Glioblastoma. Oncotarget (2015) 6:31050–68. 10.18632/oncotarget.2910 PMC4741588 25682871

[27] Juric D Castel P Griffith M Griffith OL Won HH Ellis H. Convergent Loss of PTEN Leads to Clinical Resistance to a PI(3)Kalpha Inhibitor. Nature (2015) 518:240–4. 10.1038/nature13948 PMC4326538 25409150

[28] Yan Y Li Z Zeng S Wang X Gong Z Xu Z. FGFR2-Mediated Phosphorylation of PTEN at Tyrosine 240 Contributes to the Radioresistance of Glioma. J Cell Commun Signal (2019) 13:279–80. 10.1007/s12079-019-00518-6 PMC6732132 31025173

[29] Yang JY Hung MC. A New Fork for Clinical Application: Targeting Forkhead Transcription Factors in Cance. Clin Cancer Res (2009) 15:752–7. 10.1158/1078-0432.CCR-08-0124 PMC2676228 19188143

[30] Shi ZM Wang XF Qian X Tao T Wang L Chen QD. MiRNA-181b Suppresses IGF-1R and Functions as a Tumor Suppressor Gene in Gliomas. RNA (2013) 19:552–60. 10.1261/rna.035972.112 PMC3677265 23431408

[31] Wang F Jiang H Wang S Chen B. Dual Functional MicroRNA-186-5p Targets Both FGF2 and RelA to Suppress Tumorigenesis of Glioblastoma Multiform. Cell Mol Neurobiol (2017) 37:1433–42. 10.1007/s10571-017-0474-4 PMC11482140 28213656

[32] Wang M Han Q Su Z Yu X. Transcription Factor ZNF326 Upregulates the Expression of ERCC1 and HDAC7 and Its Clinicopathologic Significance in Glioma. Lab Med (2020) 51(4):377–84. 10.1093/labmed/lmz075 31930344

[33] Kalinina OV Wichmann O Apic G Russell RB. Combinations of Protein-Chemical Complex Structures Reveal New Targets for Established Drug. PloS Comput Biol (2011) 7:e1002043. 10.1371/journal.pcbi.1002043 21573205 PMC3088657

[34] Ritchie ME Phipson B Wu D Hu Y Law CW Shi W. Limma Powers Differential Expression Analyses for RNA-Sequencing and Microarray Studies. Nucleic Acids Res (2015) 43:e47–7. 10.1093/nar/gkv007 PMC4402510 25605792

[35] Wang L Cao C Ma Q Zeng Q Wang H Cheng Z. RNA-Seq Analyses of Multiple Meristems of Soybean: Novel and Alternative Transcripts, Evolutionary and Functional Implications. BMC Plant Biol (2014) 14:169–9. 10.1186/1471-2229-14-169 PMC4070088 24939556

[36] Zhao J Wang L Hu G Wei B. A 6-Gene Risk Signature Predicts Survival of Glioblastoma Multiform. BioMed Res Int (2019) 2019:1649423–1649423. 10.1155/2019/1649423 31531345 PMC6720050

[37] Eng KH Schiller E Morrell K. On Representing the Prognostic Value of Continuous Gene Expression Biomarkers With the Restricted Mean Survival Curve. Oncotarget (2015) 6:36308–18. 10.18632/oncotarget.6121 PMC4742179 26486086

[38] Szklarczyk D Gable AL Lyon D Junge A Wyder S Huerta-Cepas J. STRING V11: Protein–Protein Association Networks With Increased Coverage, Supporting Functional Discovery in Genome-Wide Experimental Datasets. Nucleic Acids Res (2018) 47:D607–13. 10.1093/nar/gky1131 PMC6323986 30476243

[39] Zhou G Soufan O Ewald J Hancock REW Basu N Xia J. NetworkAnalyst 3.0: A Visual Analytics Platform for Comprehensive Gene Expression Profiling and Meta-Analysis. Nucleic Acids Res (2019) 47:W234–41. 10.1093/nar/gkz240 PMC6602507 30931480

[40] Shannon P Markiel A Ozier O Baliga NS Wang JT Ramage D. Cytoscape: A Software Environment for Integrated Models of Biomolecular Interaction Network. Genome Res (2003) 13:2498–504. 10.1101/gr.1239303 PMC403769 14597658

[41] Dhillon AS Hagan S Rath O Kolch W. MAP Kinase Signalling Pathways in Cancer. Oncogene (2007) 26:3279–90. 10.1038/sj.onc.1210421 17496922

[42] Shirahata M Iwao-Koizumi K Saito S Ueno N Oda M Hashimoto N. Gene Expression-Based Molecular Diagnostic System for Malignant Gliomas Is Superior to Histological Diagnosi. Clin Cancer Res (2007) 13:7341–56. 10.1158/1078-0432.CCR-06-2789 18094416

[43] Vauléon E Tony A Hamlat A Etcheverry A Chiforeanu DC Menei P. Immune Genes Are Associated With Human Glioblastoma Pathology and Patient Survival. BMC Med Genomics (2012) 5. 10.1186/1755-8794-5-41 PMC3507656 22980038

[44] Doucette T Rao G Rao A Shen L Aldape K Wei J. Immune Heterogeneity of Glioblastoma Subtypes: Extrapolation From the Cancer Genome Atlas. Cancer Immunol Res (2013) 1:112–22. 10.1158/2326-6066.CIR-13-0028 PMC3881271 24409449

[45] Wood SL Pernemalm M Crosbie PA Whetton AD. The Role of the Tumor-Microenvironment in Lung Cancer-Metastasis and Its Relationship to Potential Therapeutic Targets. Cancer Treat Rev (2014) 40:558–66. 10.1016/j.ctrv.2013.10.001 24176790

[46] Quail DF Joyce JA. Microenvironmental Regulation of Tumor Progression and Metastasis. Nat Med (2013) 19:1423–37. 10.1038/nm.3394 PMC3954707 24202395

[47] Bussard KM Mutkus L Stumpf K Gomez-Manzano C Marini FC. Tumor-Associated Stromal Cells as Key Contributors to the Tumor Microenvironment. Breast Cancer Res (2016) 18(1):84. 10.1186/s13058-016-0740-2 27515302 PMC4982339

[48] Mollaoglu G Jones A Wait SJ Mukhopadhyay A Jeong S Arya R. The Lineage-Defining Transcription Factors SOX2 and NKX2-1 Determine Lung Cancer Cell Fate and Shape the Tumor Immune Microenvironmen. Immunity (2018) 49:764–779.e769. 10.1016/j.immuni.2018.09.020 30332632 PMC6197489

[49] Mascaux C Angelova M Vasaturo A Beane J Hijazi K Anthoine G. Immune Evasion Before Tumour Invasion in Early Lung Squamous Carcinogenesis. Nature (2019) 571:570–5. 10.1038/s41586-019-1330-0 31243362

[50] Yoshihara K Shahmoradgoli M Martínez E Vegesna R Kim H Torres–Garcia W. Inferring Tumour Purity and Stromal and Immune Cell Admixture From Expression Data. Nat Commun (2013) 4:2612. 10.1038/ncomms3612 24113773 PMC3826632

[51] Bi K-W Wei X-G Qin X-X Li B. BTK Has Potential to Be a Prognostic Factor for Lung Adenocarcinoma and an Indicator for Tumor Microenvironment Remodeling: A Study Based on TCGA Data Minin. Front Oncol (2020) 10:424–4. 10.3389/fonc.2020.00424 PMC7175916 32351880

[52] Zhang C Cheng W Ren X Wang Z Liu X Li G. Tumor Purity as an Underlying Key Factor in Gliom. Clin Cancer Res (2017) 23:6279–91. 10.1158/1078-0432.CCR-16-2598 28754819

[53] Chen P Zhao D Li J Liang X Li J Chang A. Symbiotic Macrophage-Glioma Cell Interactions Reveal Synthetic Lethality in PTEN-Null Gliom. Cancer Cell (2019) 35:868–884 e866. 10.1016/j.ccell.2019.05.003 31185211 PMC6561349

[54] 7Parsa AT Waldron JS Panner A Crane CA Parney IF Barry JJ. Loss of Tumor Suppressor PTEN Function Increases B7-H1 Expression and Immunoresistance in Glioma. Nat Med (2007) 13:84–8. 10.1038/nm1517 17159987

[55] Xia Q Zhang H Zhang P Li Y Xu M Li X. Oncogenic Smurf1 Promotes PTEN Wild-Type Glioblastoma Growth by Mediating PTEN Ubiquitylation. Oncogene (2020) 39:5902–15. 10.1038/s41388-020-01400-1 32737433

[56] Gatti M Pattarozzi A Würth R Barbieri F Florio T. Somatostatin and Somatostatin Receptors 1, 2 and 5 Selective Agonists Inhibit C6 Glioma Cell Growth *In Vitro* and *In Vivo*: Analysis of Activated Intracellular Pathways. Regul Peptides (2010) 164:38–8. 10.1016/j.regpep.2010.07.095

[57] Qu J Yan R Chen J Xu T Zhou J Wang M. HMGN5: A Potential Oncogene in Gliomas. J Neurooncol (2011) 104:729–36. 10.1007/s11060-011-0558-9 21373965

[58] Li G Wang Z Zhang C Liu X Yang F Sun L. MEGF10, a Glioma Survival-Associated Molecular Signature, Predicts IDH Mutation Statu. Dis Markers (2018) 2018:5975216. 10.1155/2018/5975216 29887919 PMC5985127

[59] Zheng J Zhang M Zhang L Ding X Li W Lu S. HSPC159 Promotes Proliferation and Metastasis by Inducing Epithelial-Mesenchymal Transition and Activating the PI3K/Akt Pathway in Breast Cancer. Cancer Sci (2018) 109:2153–63. 10.1111/cas.13631 PMC6029831 29737572

[60] Dazhi W Jing D Chunling R Mi Z Zhixuan X. Elevated SLC6A6 Expression Drives Tumorigenesis and Affects Clinical Outcomes in Gastric Cancer Biomark Med (2019) 13(2):95–104. 10.2217/bmm-2018-0256 30767502

[61] Pei Y-F Liu J Cheng J Wu W-D Liu X-Q. Silencing of LAMC2 Reverses Epithelial-Mesenchymal Transition and Inhibits Angiogenesis in Cholangiocarcinoma *via* Inactivation of the Epidermal Growth Factor Receptor Signaling Pathway. Am J Pathol (2019) 189:1637–53. 10.1016/j.ajpath.2019.03.012 31345467

[62] Gong X Carmon KS Lin Q. LGR6 Is a High Affinity Receptor of R-Spondins and Potentially Functions as a Tumor Suppressor. PloS One (2012) 7. 10.1371/journal.pone.0037137 PMC3355120 22615920

[63] Kim JW Marquez CP Kostyrko K Koehne AL Marini K Simpson DR. Antitumor Activity of an Engineered Decoy Receptor Targeting CLCF1-CNTFR Signaling in Lung Adenocarcinoma. Nat Med (2019) 25:1783–95. 10.1038/s41591-019-0612-2 PMC7087454 31700175

[64] Chang N Ahn SH Kong DS Lee HW Nam DH. The Role of STAT3 in Glioblastoma Progression Through Dual Influences on Tumor Cells and the Immune Microenvironment. Mol Cell Endocrinol (2017) 451:53–65. 10.1016/j.mce.2017.01.004 28089821

[65] Moon SH Kim DK Cha Y Jeon I Song J Park KS. PI3K/Akt and Stat3 Signaling Regulated by PTEN Control of the Cancer Stem Cell Population, Proliferation and Senescence in a Glioblastoma Cell Line. Int J Oncol (2013) 42:921–8. 10.3892/ijo.2013.1765 23314408

[66] Sinha S Renganathan A Nagendra PB Bhat V Mathew BS Rao MRS. AEBP1 Down Regulation Induced Cell Death Pathway Depends on PTEN Status of Glioma Cells. Sci Rep (2019) 9:14577. 10.1038/s41598-019-51068-1 31601918 PMC6787275

[67] Majdalawieh AF Massri M Ro H-S. AEBP1 is a Novel Oncogene: Mechanisms of Action and Signaling Pathway. J Oncol (2020) 2020:1–20. 10.1155/2020/8097872 PMC7273425 32565808

[68] Hu W Jin L Jiang CC Long GV Scolyer RA Wu Q. AEBP1 Upregulation Confers Acquired Resistance to BRAF (V600E) Inhibition in Melanoma. Cell Death Dis (2013) 4. 10.1038/cddis.2013.441 PMC3847319 24201813

[69] Xing Y Zhang Z Chi F Zhou Y Ren S Zhao Z. AEBP1, a Prognostic Indicator, Promotes Colon Adenocarcinoma Cell Growth and Metastasis Through the NF-κb Pathway. (2019) 58: (10):1795–808. 10.1002/mc.23066 31219650

[70] Yorozu A Yamamoto E Niinuma T Tsuyada A Maruyama R Kitajima H. Upregulation of AEBP1 in Endothelial Cells Promotes Tumor Angiogenesis in Colorectal Cancer. Cancer Sci (2020) 111(5):1631–44. 10.1111/cas.14360 PMC7226196 32086986

[71] Kim H Bhattacharya A Qi L. Endoplasmic Reticulum Quality Control in Cancer: Friend or Foe. Semin Cancer Biol (2015) 33:25–33. 10.1016/j.semcancer.2015.02.003 25794824 PMC4523434

[72] Su YA Hutter CM Trent JM Meltzer PS. Complete Sequence Analysis of a Gene (OS-9) Ubiquitously Expressed in Human Tissues and Amplified in Sarcomas. Mol Carcinogenesis (1996) 15:270–5. 10.1002/(SICI)1098-2744(199604)15:4<270::AID-MC4>3.0.CO;2-K 8634085

[73] Gleadle JH Mahon PC Oh J Kelly B Krishnamachary B Pearson M. OS-9 Interacts with Hypoxia-Inducible Factor 1α and Prolyl Hydroxylases to Promote Oxygen-Dependent Degradation of HIF-1α. Mol Cell (2005) 17(4):503–12. 10.1016/j.molcel.2005.01.011 15721254

[74] Müller S Brown PJ. Epigenetic Chemical Probe. Clin Pharmacol Ther (2012) 92:689–93. 10.1038/clpt.2012.154 23093316

[75] Li Z Guo J Wu Y Zhou Q. The BET Bromodomain Inhibitor JQ1 Activates HIV Latency Through Antagonizing Brd4 Inhibition of Tat-Transactivation. Nucleic Acids Res (2012) 41:277–87. 10.1093/nar/gks976 PMC3592394 23087374

[76] Matzuk MM McKeown MR Filippakopoulos P Li Q Ma L Agno JE. Small-Molecule Inhibition of BRDT for Male Contraception. Cell (2012) 150:673–84. 10.1016/j.cell.2012.06.045 PMC3420011 22901802

[77] Korb E Herre M Zucker-Scharff I Darnell RB Allis CD. BET Protein Brd4 Activates Transcription in Neurons and BET Inhibitor Jq1 Blocks Memory in Mice. Nat Neurosci (2015) 18:1464–73. 10.1038/nn.4095 PMC4752120 26301327

