# Supplementary figures and images for: Identification of the Prognostic Signatures of Glioma With Different PTEN Status
Source: Front Oncol. 2021 Jul 14;11:633357. doi: 10.3389/fonc.2021.633357 (PMC8317988; doi:10.3389/fonc.2021.633357)

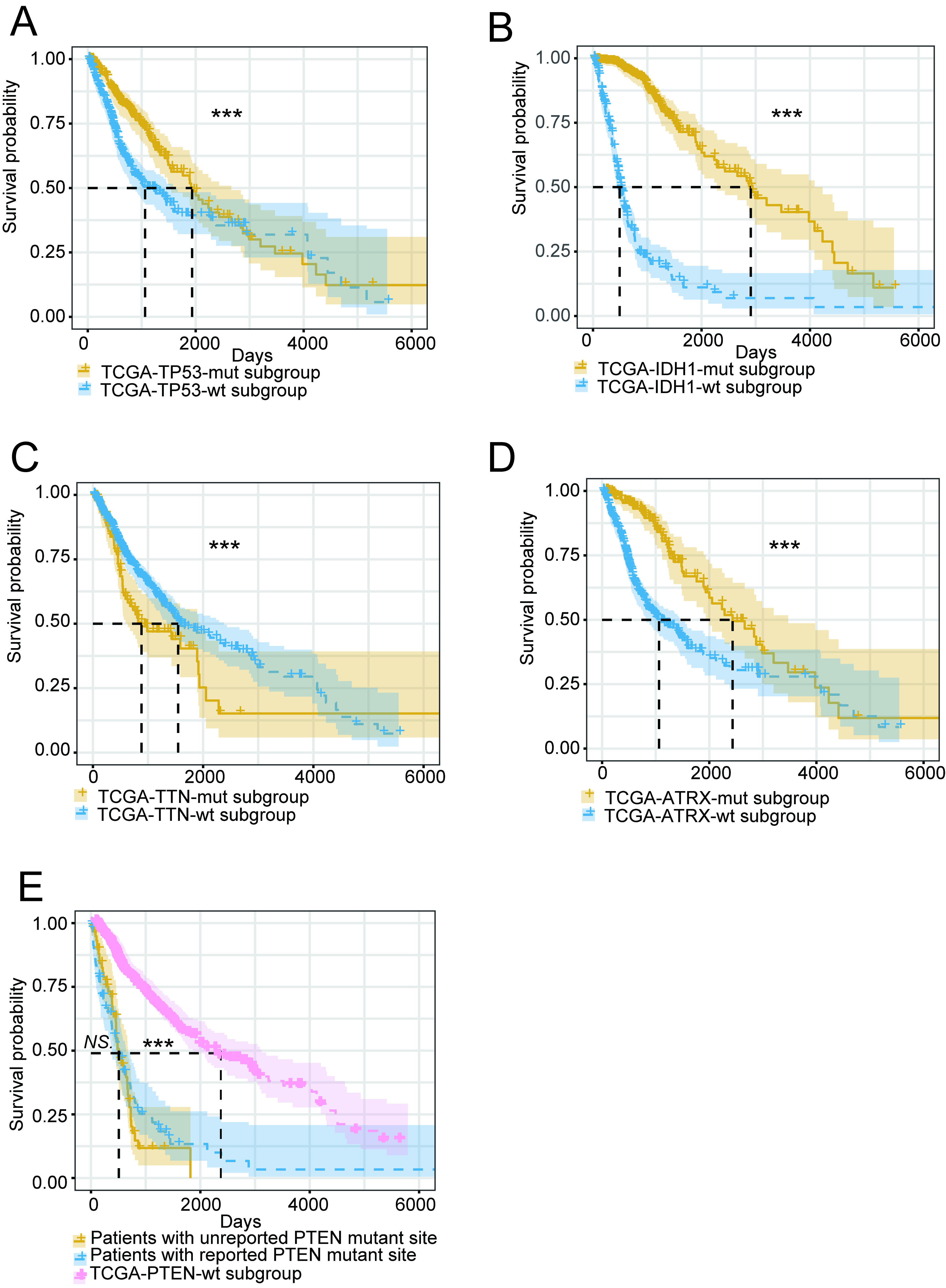

Supplement: Supplementary Figure 1 — (A) Survival analysis of TP53-wt (blue, n = 466) and TP53-mut (yellow, n = 430) in TCGA dataset; (B) Survival analysis of IDH1-wt (blue, n = 481) and IDH1-mut (yellow, n = 415) in TCGA dataset; (C) Survival analysis of TTN-wt (blue, n = 531) and TTN-mut (yellow, n = 365) in TCGA dataset; (D) Survival analysis of ATRX-wt (blue, n = 630) and ATRX-mut (yellow, n = 266) in TCGA dataset; (E) Survival analysis of PTEN-wt (pink, n = 733), patients with unreported PTEN mutant sites (yellow, n = 70) and patients with reported PTEN mutant sites (blue, n = 88) in TCGA dataset. [file Image_1.tif]

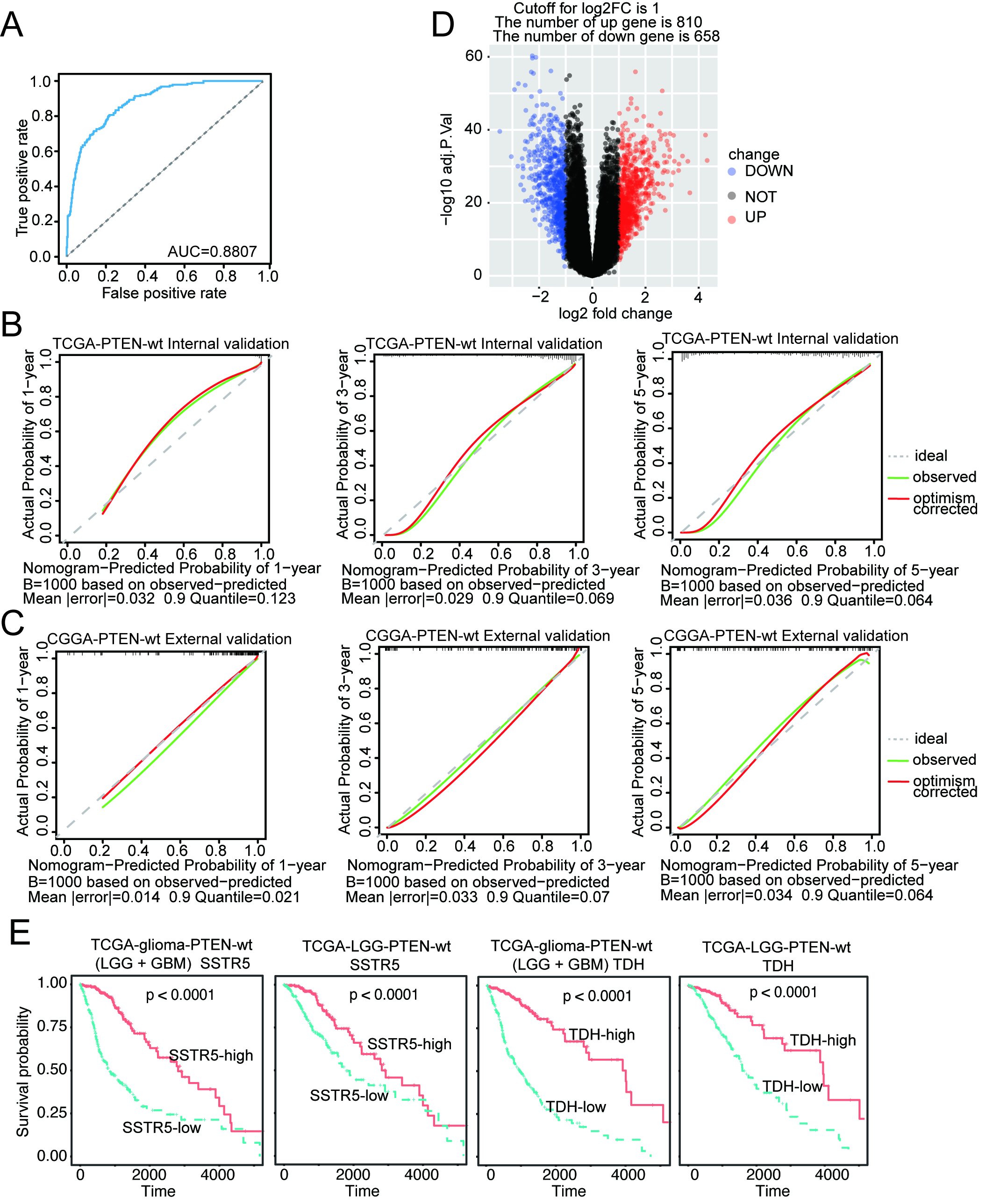

Supplement: Supplementary Figure 2 — (A) ROC curve of L1-Penalized (LASSO) Cox-pH regression model in TCGA PTEN-wt subgroup; (B) The calibration plots by internal validation of Bootstrap Resamples (1000) in TCGA-PTEN-wt dataset; (C) The calibration plots by external validation of CGGA PTEN-wt dataset (n = 135); (D) The volcano plot of DEGs between high-risk patients and low-risk patients in TCGA PTEN-wt subgroup (DGEs: n = 1468); (E) The decrease expression of SSTR5 and TDH results in a poor prognosis in PTEN-wt patients. [file Image_2.tif]

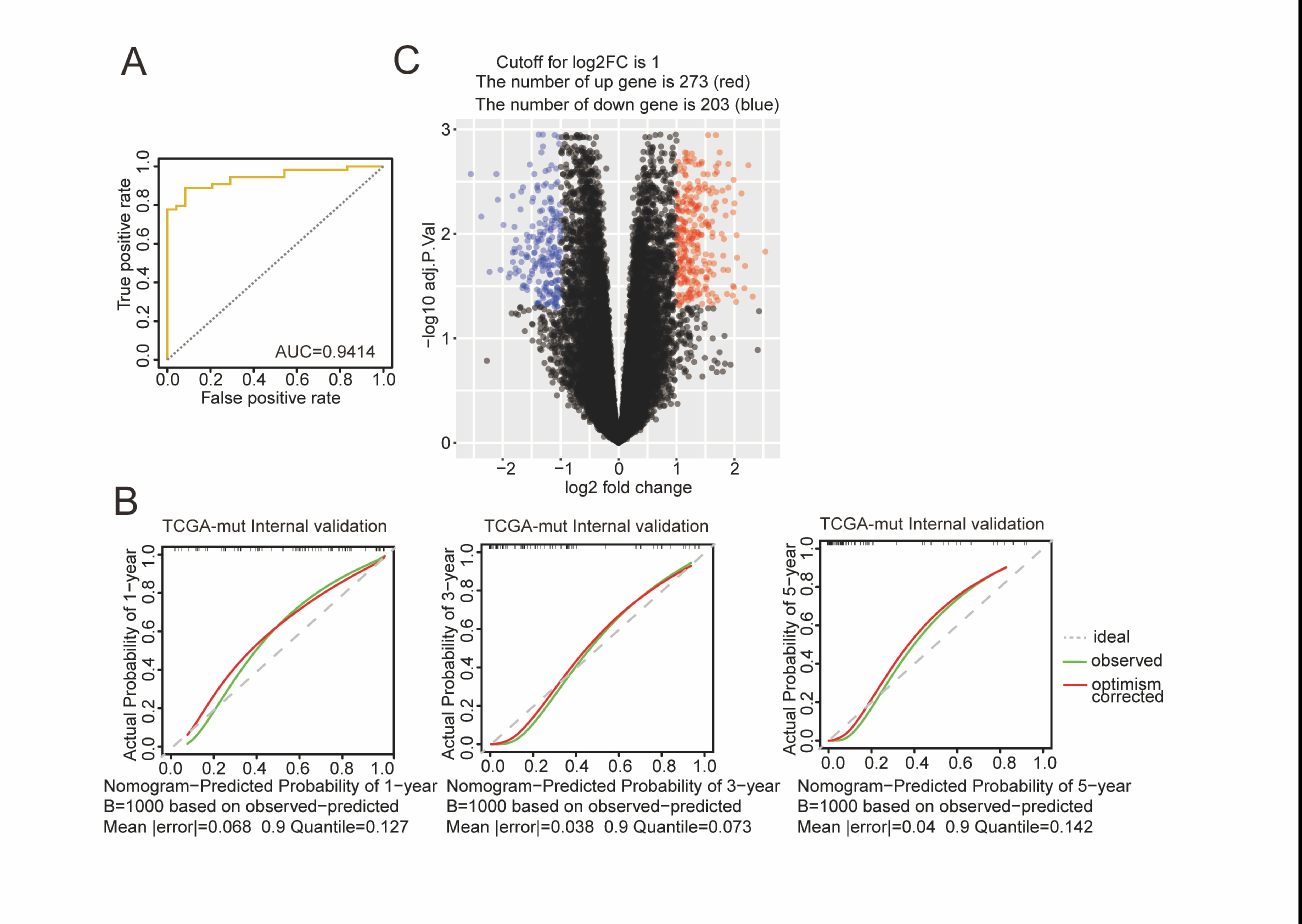

Supplement: Supplementary Figure 3 — (A) ROC curve of L1-Penalized (LASSO) Cox-pH regression model in TCGA PTEN-mut subgroup; (B) The calibration plots by internal validation of Bootstrap Resamples (1000) in TCGA-PTEN-mut dataset; (C) The volcano plot of DEGs between high-risk score patients and low-risk score patients in TCGA PTEN-mut subgroup (DGEs: n = 476). [file Image_3.jpg]

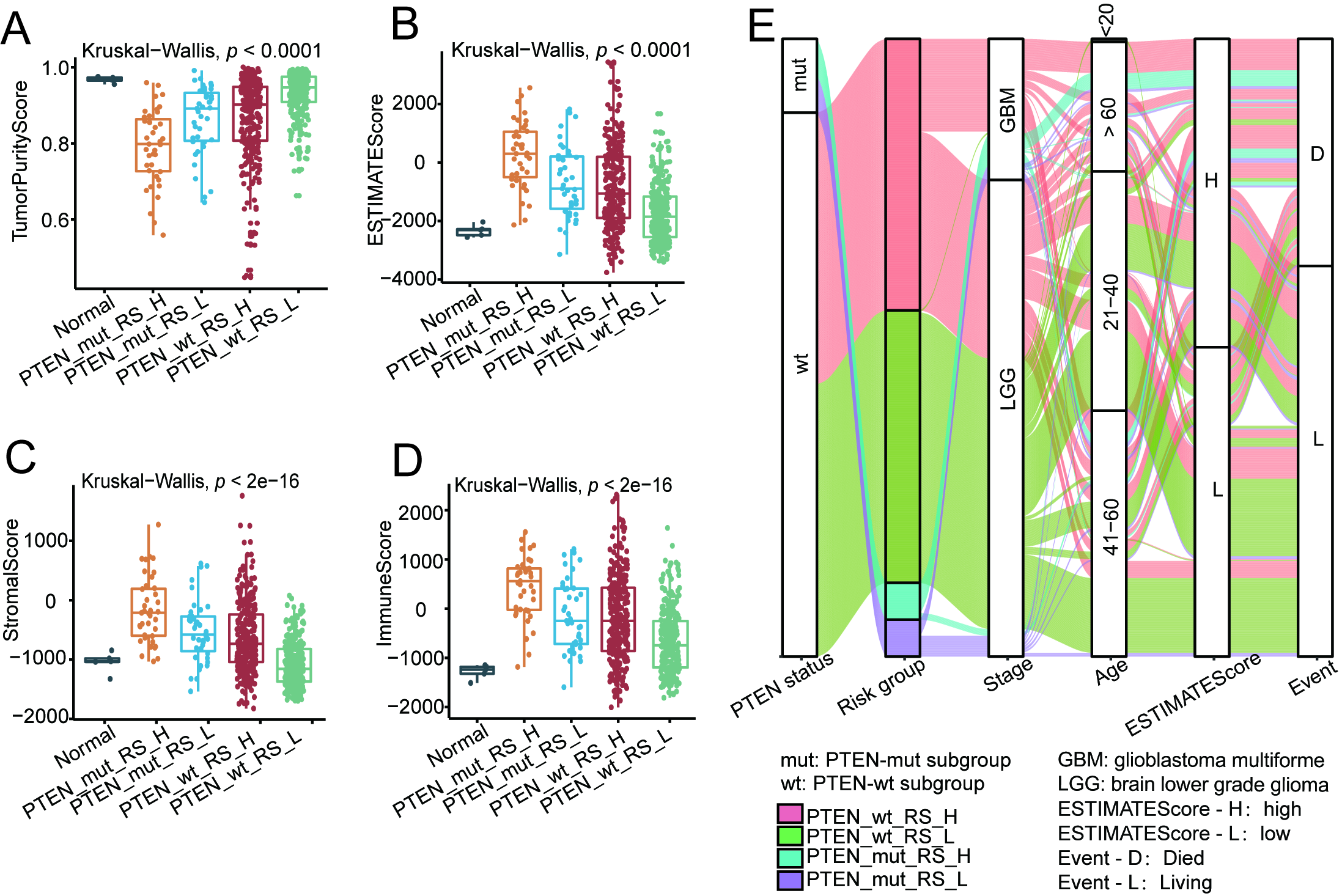

Supplement: Supplementary Figure 4 — The correlation between risk score with malignancy of glioma: (A–D) TumorPurityScore (A), ESTIMATEScore (B), StromalScore (C), and ImmuneScore (D) in patients with a different risk score, including high-risk score patients in TCGA PTEN-mut subgroup (PTEN_mut_RS_H), low-risk score patients in TCGA PTEN-mut subgroup (PTEN_mut_RS_H), high-risk score patients in TCGA PTEN-wt subgroup (PTEN_wt _RS_H), and low-risk score patients in TCGA PTEN-mut subgroup (PTEN_wt _RS_L); (E) The alluvial diagram of TCGA glioma dataset, each line represents a patient. [file Image_4.tif]

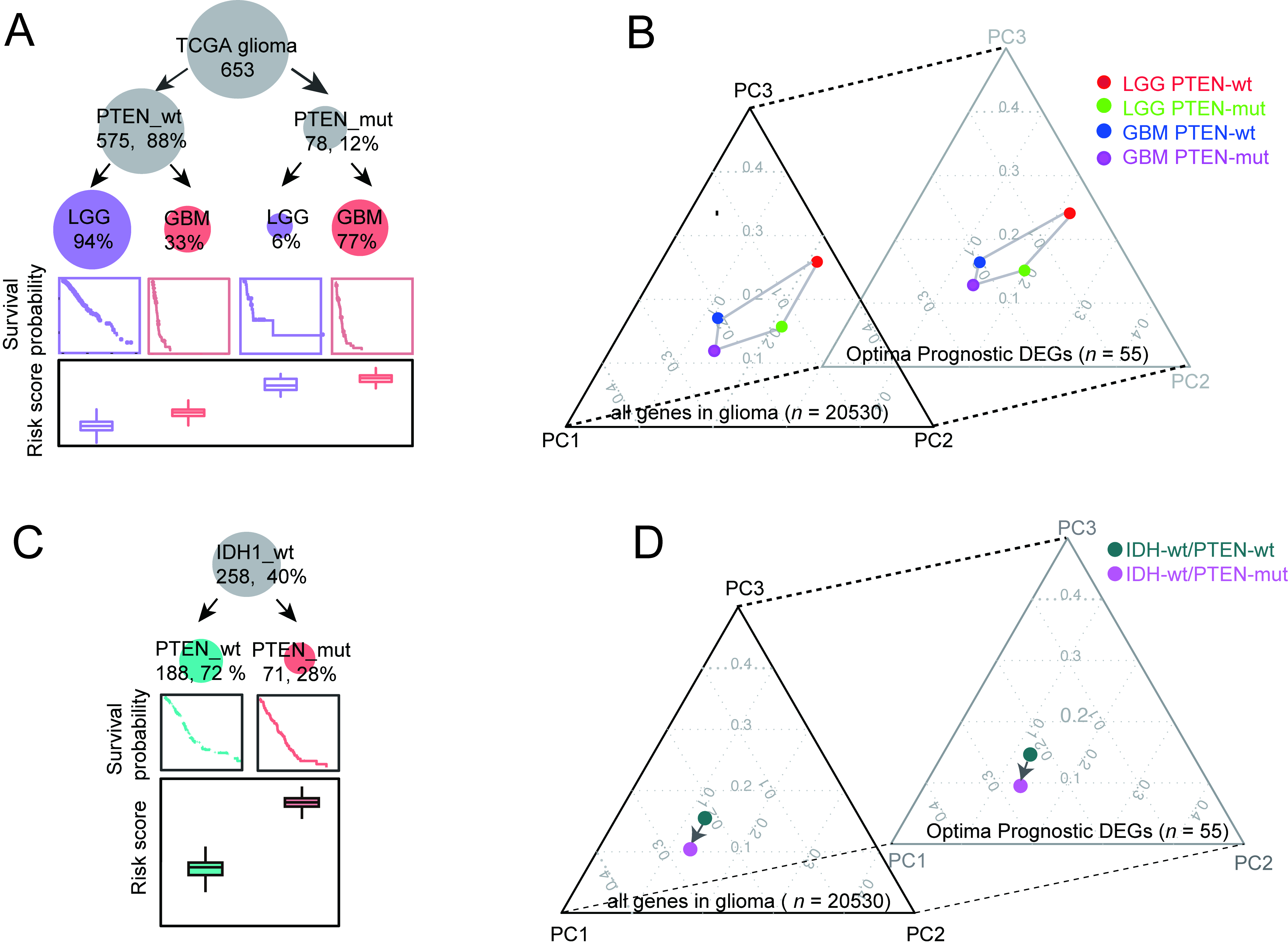

Supplement: Supplementary Figure 5 — (A) The number of patients, survival rates, and the risk score established by our model in different types of TCGA glioma dataset; (B) PCA plots of different type of glioma by the expression of all genes and Optimal Prognostic DEGs (OPR-DEGs in PTEN-wt and PTEN-mut); (C) The number of patients, survival rates, and the risk score established by our model in IDH-wt glioma; (D) PCA plots of different type of IDH-wt glioma by the expression of all genes and Optimal Prognostic DEGs (OPR-DEGs in PTEN-wt and PTEN-mut, Supplementary Tables 5 and 7 ). [file Image_5.tif]

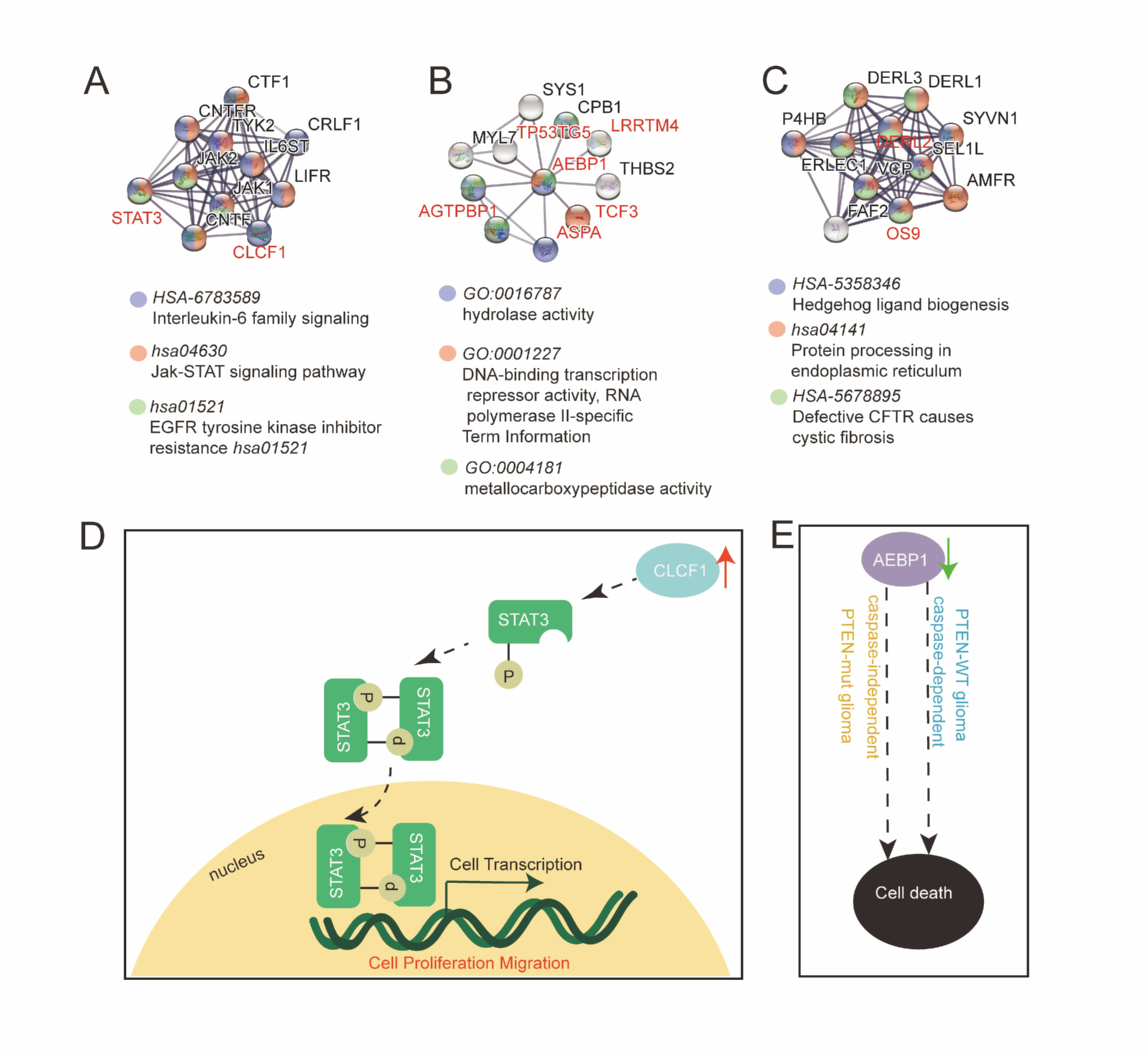

Supplement: Supplementary Figure 6 — (A–C) The protein-protein (PPI) network of CLCF1 (A), AEBP1 (B), and OS9 (C); (D) The mechanism of cell proliferation induced by CLCF1; (E) The mechanism of cell death induced by AEBP1 downregulation. [file Image_6.jpg]
